# Cerebellar functional connectivity change is associated with motor and neuropsychological function in early stage drug-naïve patients with Parkinson’s disease

**DOI:** 10.3389/fnins.2023.1113889

**Published:** 2023-06-22

**Authors:** Li Jiang, Jiachen Zhuo, Andrew Furman, Paul S. Fishman, Rao Gullapalli

**Affiliations:** ^1^Department of Diagnostic Radiology & Nuclear Medicine, University of Maryland School of Medicine, Baltimore, MD, United States; ^2^Center for Advanced Imaging Research (CAIR), University of Maryland School of Medicine, Baltimore, MD, United States; ^3^Department of Neurology, University of Maryland School of Medicine, Baltimore, MD, United States

**Keywords:** Parkinson’s disease, functional connectivity, resting-state fMRI, cerebellum, neuropsychological function

## Abstract

**Introduction:**

Parkinson’s Disease (PD) is a progressive neurodegenerative disorder affecting both motor and cognitive function. Previous neuroimaging studies have reported altered functional connectivity (FC) in distributed functional networks. However, most neuroimaging studies focused on patients at an advanced stage and with antiparkinsonian medication. This study aims to conduct a cross-sectional study on cerebellar FC changes in early-stage drug-naïve PD patients and its association with motor and cognitive function.

**Methods:**

Twenty-nine early-stage drug-naïve PD patients and 20 healthy controls (HCs) with resting-state fMRI data and motor UPDRS and neuropsychological cognitive data were extracted from the Parkinson’s Progression Markers Initiative (PPMI) archives. We used seed-based resting-state fMRI (rs-fMRI) FC analysis and the cerebellar seeds were defined based on the hierarchical parcellation of the cerebellum (AAL atlas) and its topological function mapping (motor cerebellum and non-motor cerebellum).

**Results:**

The early stage drug-naïve PD patients had significant differences in cerebellar FC when compared with HCs. Our findings include: (1) Increased intra-cerebellar FC within motor cerebellum, (2) increase motor cerebellar FC in inferior temporal gyrus and lateral occipital gyrus within ventral visual pathway and decreased motor-cerebellar FC in cuneus and dorsal posterior precuneus within dorsal visual pathway, (3) increased non-motor cerebellar FC in attention, language, and visual cortical networks, (4) increased vermal FC in somatomotor cortical network, and (5) decreased non-motor and vermal FC within brainstem, thalamus and hippocampus. Enhanced FC within motor cerebellum is positively associated with the MDS-UPDRS motor score and enhanced non-motor FC and vermal FC is negatively associated with cognitive function test scores of SDM and SFT.

**Conclusion:**

These findings provide support for the involvement of cerebellum at an early stage and prior to clinical presentation of non-motor features of the disease in PD patients.

## 1. Introduction

Parkinson’s disease (PD) is the second most common neurodegenerative disorder and the most common movement disorder in elderly population with increasing prevalence from about 1% at age 60 to 4% by age 80 worldwide (Elbaz et al., 2016; Tysnes and Storstein, 2017; Marras, Beck et al., 2018; Bjornevik et al., 2020). PD is often characterized by progressive deterioration of motor function including resting tremor, bradykinesia, rigidity, and postural instability. PD is also involved in non-motor dysfunctions including cognitive impairment, mood disorders, impaired olfaction, hallucination, etc (Jankovic, 2008).

Resting-state functional MRI (rs-fMRI), as a non-invasive and task-free tool, has been used to investigate the functional abnormalities and has provided important insights on the pathophysiological mechanisms of various neurodegenerative diseases (Szymanski et al., 2010; Ehrlich and Walker, 2017; Annavarapu et al., 2019; Manjaly et al., 2019), including PD (Wu et al., 2009; Hacker et al., 2012; Ma et al., 2017; Tahmasian et al., 2017; Tuite, 2017; Hou et al., 2018; Tuovinen et al., 2018; Filippi et al., 2019; Tessitore et al., 2019). Given that many of the motor symptoms in PD can be attributed to the loss of dopaminergic neuron in the SNpc (Damier et al., 1999), most rs-fMRI studies have focused on the striato-thalamo-cortical pathways. However, several key features of PD, such as non-motor symptoms and the heterogeneity of the disease, cannot be explained adequately by basal ganglia dysfunction alone and is an active area of research (Manza et al., 2016; Park et al., 2019). More recently, emerging evidence from multiple converging modalities (anatomical, clinical, pathophysiological and neuroimaging) have shown that the cerebellum may contribute substantially to the motor and non-motor symptoms of PD (Yu et al., 2007; Wu and Hallett, 2013; Mirdamadi, 2016; Caligiore et al., 2017; Starowicz-Filip et al., 2017; Schmahmann, 2019). Neuroanatomic and functional studies provided evidences that distinct cerebellum sub-regions receive information from widespread neocortical areas, including portions of the frontal, parietal, temporal, and occipital lobes, while the efferent from the cerebellar nuclei projects to multiple subdivisions of the thalamus, which, in turn, project to a myriad of neocortical areas, including premotor, prefrontal, and posterior parietal areas of the cerebral cortex (Bostan et al., 2013). Moreover, recent findings have shown that the cerebellum and basal ganglia are densely interconnected (Caligiore et al., 2017). Given its anatomical and functional inter-connections with the basal ganglia and much of the cortical cortex, cerebellum not only has influence on motor function, but may also influence a range of higher-order non-motor functions (Bostan et al., 2013; Caligiore et al., 2017).

Previous rs-fMRI studies have reported altered cerebellar FC in PD patients and its association with motor and cognitive test scores (Wu and Hallett, 2013; O'Callaghan et al., 2016; Kaut et al., 2020; Maiti et al., 2020, 2021). Altered FC between the cerebellum and large-scale cortical networks, including the sensorimotor, dorsal attention, default networks, and frontoparietal network, have been observed in PD patients compared to normal controls (O'Callaghan et al., 2016; Kaut et al., 2020). However, most of the rs-fMRI studies of cerebellum have focused on patients with moderate or advanced stages of PD who were treated with anti-parkinsonian medication. Acute and chronic use of levodopa has been shown to modify the PD-induced changes in local neural activity and FC in patients with PD (Buhmann et al., 2003; Hershey et al., 2003; Black et al., 2005; Copland et al., 2009; Maillet et al., 2012; Esposito et al., 2013; Tahmasian et al., 2015; O'Callaghan et al., 2016; Kaut et al., 2020). As such, it is often unclear whether the results of these PD studies are due to the disease, medication being used, or a combination of both factors. Moreover, only very few studies reported that altered cerebellar FC and FC between the “motor” cerebellum and SMA was positively correlated with motor scores, while intra-cerebellar connectivity was positively correlated with cognitive scores in PD patients with cognitive impairment (Dan et al., 2021). It becomes important to understand the underlying pathophysiological mechanisms of PD at an early stage and without medication administration.

In this study, we aimed to study the change of cerebellar FC in early stage drug-naïve PD patients. In particular, we sought to determine the specific pattern of FC changes that occur across the motor, non-motor, and vermis regions of the cerebellum in order to provide insight into the neurophysiological mechanisms that may, independently from treatment-related side effects, generate the key motor and cognitive symptoms of Parkinson’s Disease. We used the anatomic atlas of cerebellum as seed regions and performed seed-based rs-fMRI analysis to investigate FC changes between cerebellar sub-regions and cortical and subcortical brain regions. We also evaluated the association of cerebellar FC with motor and cognitive function assessments among the PD patients.

## 2. Methods and materials

### 2.1. Participants

Data used in the preparation of this article were obtained from the Parkinson’s Progression Markers Initiative (PPMI) database^1^ and only early stage drug-naïve PD patients with available rs-fMRI data were included in this study. Initially, we identified 102 PD patients who had one or more rs-fMRI scans. Among them, however, only 33 PD patients (20 males and 13 females, aged between 38 ~ 75 years old, and 11 ~ 22 years of education) obtained at least one rs-fMRI scan before the initiation of PD treatment. All the 33 PD patients had at least two of the following symptoms: resting tremor, bradykinesia, rigidity, or either asymmetric resting tremor or asymmetric bradykinesia and at Hoehn and Yahr Stage I or II (without impairment of posture balance). These PD patients were normal or mild cognitive impairment (MCI) assessed by Global Cognitive Test of Montreal Cognitive Assessment (MoCA). The cutoff MoCA score to differentiate normal cognition and MCI was set as 26. No patients had any atypical PD syndromes due to drugs or metabolic disorders, encephalitis, or other neurodegenerative diseases. A cohort of 22 age-and education-matched HC participants (17 males and 5 females, aged between 40 and 79 years old, and 13–22 years of education) were extracted from PPMI too. For both PD patients and HC participants, the first available rs-fMRI data was used.

### 2.2. MDS-unified Parkinson’s disease rating scale test for motor function

The Movement Disorder Society-sponsored revision of the Unified Parkinson’s Disease Rating Scale (MDS-UPDRS) is used to evaluate the severity and progression of PD among patients (Goetz, Tilley et al., 2008). The MDS-UPDRS Part III Motor Score provides a comprehensive evaluation of motor function covering speech, facial expression, muscle rigidity, tremor (rest, posture, and kinetic), gait, posture instability, etc. It includes 18 items resulting in 33 scores by location and lateralization. Each score has five response options, ranging from 0 ~ 4 representing normal, slight, mild, moderate, and severe. The maximum of the sum of all the scores is 132. Higher score indicates more severe impairment.

### 2.3. Neuropsychological cognitive function test

The major cognitive function test was conducted using Global Cognitive Test of Montreal Cognitive Assessment (MoCA). In addition, a comprehensive neuropsychological battery assessment in five cognitive domains was also administrated: (1) Hopkins Vernal Learning Test-Revised (HVLT) for verbal memory, (2) Benton Judgment of Line Orientation (BJLO, using one of the two short form with 15 items) for visuospatial function, (3) Semantic Fluency Tests (SFT) including animal, vegetable and fruit categories for executive function, (4) WMS-III Letter-Number Sequencing Test (LNST) for working memory, and (5) Symbol Digit Modalities Test (SDM) for attention, perceptual speed, motor speed, and visual scanning. In this study, the assessment time of the used cognitive scores were marked as the same as that of the used rs-fMRI data (the recording time difference between the assessment and imaging were about ±1 month).

### 2.4. MRI data acquisition

Standardized MRI protocols were used for brain structural and functional scans on 3 T Siemens Tim Trio MR scanner. The high resolution isotropic 3D T1 structural images were acquired in sagittal orientation using a MPRAGE GRAPPA protocol and imaging parameters of repetition time (TR) = 2,300 ms, echo time (TE) = 2.98 ms, flip angle (FA) = 9^o^, field of view (FOV) = 240 × 256 mm^2^, and voxel size = 1 × 1 × 1 mm^3^. The rs-fMRI BOLD images were acquired in axial direction using a T2* weighted single shot EPI sequence with imaging parameters of TR = 2,400 ms, TE = 25 ms, FA = 80^o^, FOV = 222 × 222 mm^2^, voxel size = 3.294 × 3.294 × 3.3 mm^3^, with a total of 210 EPI volumes. The participants were instructed to remain quiet, with their eyes open and not fall asleep throughout the rs-fMRI scan.

### 2.5. MRI data preprocessing and denoising

The structural MRI and resting-state fMRI (rs-fMRI) data were preprocessed using the CONN toolbox (version 18) (http://www.nitrc.org/projects/conn, RRID: SCR_009550). The preprocessing steps for the structural T1-MPRAGE images involved normalization to the MNI152 template space with a spatial resolution of 1x1x1 mm^3^ and simultaneous segmentation into gray matter (GM), white matter (WM), and cerebral spinal fluid (CSF) using the prior tissue probability distribution map (Ashburner and Friston, 2007).

For the rs-fMRI data, the preprocessing steps included motion estimation and correction through realignment and unwarp, slice timing correction, artifact detection using the ART method, simultaneous segmentation of GM/WM/CSF, normalization to the standard MNI template with a resolution of 2x2x2 mm^3^, and spatial smoothing with a 6 mm full-width at half-maximum (FWHM) Gaussian kernel. During the artifact detection preprocessing step, quality control (QC) timeseries were computed, including Global Signal Change (GSC) and Framewise Displacement (FD). These QC measures were used to assess and monitor the data quality and the impact of motion-related artifacts (Morfini et al., 2023). The outlier volumes were identified for scubbing.

Further denoising steps included linear regression, band filtering (0.01–0.1 Hz), and detrending to minimize the presence of non-neural noise and residual head motion effects in the rs-fMRI BOLD signal. The confounds for linear regression included the first five principal components of the average WM and CSF signals, Friston24 motion parameters (six motion realignment parameters and their first-order derivatives, the six quadratic motion realignment parameters and their first-order derivatives), QC time series, and Scrubbing. We also setup realignment measures, QC timeseresis and Scrubbing for further remove motion effect on BOLD signal. The outlier volumes were removed from further FC analysis.

Subsequently, we conducted quality checking as detailed in (Morfini et al., 2023). We also visually inspected the normalized rs-fMRI data specifically for full cerebellum coverage. As a result, only 29 PD patients and 20 HCs were included in the subsequent cerebellar FC analysis.

### 2.6. Cerebellum seed ROIs

**Figure 1:**
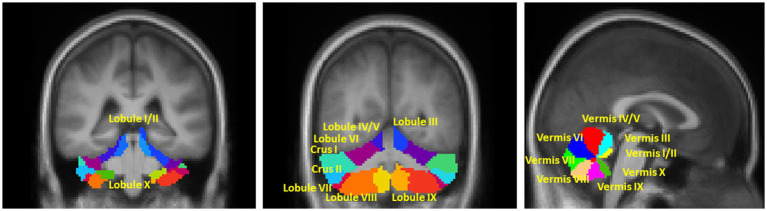
Illustration of the seed ROIs in **(A,B)** motor and non-motor hemispherical cerebellum; **(C)** Vermis. The right and left lobule ROIs were analyzed separately. The mask of all ROIs were extracted from the AAL atlas which was based on the anatomic parcellation of the human cerebellum (Diedrichsen et al., 2009).

The Conn toolbox provides a cerebellum parcellation based on the AAL atlas (Diedrichsen et al., 2009), which includes 26 subregions, such as lobular III, IV/V, VI, Crus I, Crus II, VIIb, VIII, IX, and X, as well as vermis I/II, III, IV/V, VI, VII, VIII, IX, and X. However, as the parcellation is based on young healthy population, we manually modified the cerebellar subregions by overlapping them on the averaged normalized structural and resting-state fMRI data to account for cerebellar atrophy in our older population. The final modified subregions, which were overlapped on the averaged normalized T1mprage in this study, are shown in Figure 1. Considering previous evidence that the left and right cerebellar hemispheres have distinct functions and connection to lateralized association cortex (Stoodley and Schmahmann 2009; Stoodley and Schmahmann, 2010), we kept the lobular seed ROIs by hemispheres to explore potential lateral differences. Additionally, the vermis seed ROIs were also segregated from lobular seeds since studies have shown that the cerebellar vermis has plays a distinct role in PD (Wu and Hallett, 2013). Therefore, a total of 28 cerebellar seed ROIs were used in this study.

According to topographic function mapping of the human cerebellum, it can be functionally divided into double representations of the motor cerebellum (first = lobules IV/V/VI and second = lobule VIII) and triple representations of the non-motor cerebellum (first = lobules VI/Crus I; second = lobules Crus II/VIIB; and third = lobules IX/X) (Stoodley and Schmahmann, 2010; Buckner et al., 2011; Guell et al., 2018b; King et al., 2019). Thus, in the results section, we functionally grouped the hemispherical seed ROIs into two categories: -motor and non-motor cerebellum lobular seeds.

During the initial data processing stages, we observed strong FC between the neighboring anterior cerebellum and ventral regions of the cerebral cortex, which overshadowed the FC between the cerebellum and other cerebral regions. This effect may be due to the close physical proximity of the anterior cerebellum to ventral regions of the cerebral cortex, leading to a blurring of fMRI signal across the cerebellar-cerebral boundary (Buckner et al., 2011). This effect may also be caused by head motion even though we applied head motion controls during the preprocessing and Denoising steps. To minimize this effect, we applied an eroded cerebellum mask to confine the anterior cerebellar seeds.

### 2.7. Functional connectivity analysis

A seed-to-whole brain FC analysis was performed. The averaged time series of all the voxels within the seed ROIs was obtained from the unsmoothed rs-fMRI data to minimize contamination of the signal from neighboring lobules. FC was measured as the Pearson correlation between the averaged time series of a seed ROI and the time series of each voxel in the whole brain from spatially smoothed rs-fMRI data. Fisher z-transformation was then used to convert the correlation coefficients to normally distributed scores, in order to allow second-level general linear model (GLM) analysis. For group comparison analysis, a mixed GLM model was applied to estimate the difference of FC between the PD patients and HCs by controlling for age, gender and years of education. The significance level was set at two-sided voxel-wise uncorrected *p* < 0.005 and a cluster-size false-discovery rate (FDR) corrected *p* < 0.05 for multiple comparisons.

### 2.8. Association analysis between FC and motor and cognitive test scores

We conducted association analysis using SPSS 26 to examine the relationship between the z-value of FC in significant clusters of each seed and the total score of MDS-UPDRS III, as well as the scores from the neuropsychological tests. For clusters demonstrating significant FC differences between PD and HC participants, we calculated the average FC of voxels within each cluster. Non-parametric correlations were then performed to assess the associations between FC and MDS-UPDRS III scores, while controlling for age, gender, and education. Additionally, partial correlations were conducted between FC and cognitive test scores, with adjustments made for age, gender, and education. The significance level was set at *p* < 0.05.

### 2.9. Other statistical analysis

The group difference in motor and cognitive scores were estimated using the GLM multivariate analysis controlling for age, gender and years of education with SPSS ver26. The significant level was defined as corrected value of *p* <0.05.

## 3. Results

### 3.1. Demographic and clinical characteristic data

As shown in Table 1, a total of 33 early stage drug-naïve PD patients (20 males/13 females, mean age of 59.12 ± 10.71, range of 38 ~ 75 years old) and 22 age and education matched HC participants (17 males/5 females, mean age of 60.99 ± 10.88, range of 40 ~ 79 years old) from the PPMI archives were included in this study. Four patients were excluded for FC analysis due to partial cerebellar coverage. Over 85% of the PD and HC participants were right-hand dominant. For PD patients, about 67% had right-side motor onset. About 97% were at early stage (under Hoehn-Yahr stage 3) with mild symptoms of rest tremor, rigidity, and bradykinesia. At the initial visit, there was no significant group difference in cognitive function MoCA score between the PD patients and HC participants (PD: 27.30 ± 2.48, HC: PD: 28.18 ± 1.10; *p* = 0.125). Furthermore, 25 PD patients and 20 HC participants had normal cognitive function with MoCA score greater than 26 (mean MoCA of PD = 28.52 and HC = 27.95) and only 8 PD patients and 2 HC participants had mild cognition impairment (MCI) (mean MoCA of PD = 23.5 and HC = 24).

**Table 1:** Demographics and Clinical characteristics for PD patients and HCs.

Variables		HCs	PD Patients	*p*
*N*		22	33	
Age at rsfMRI (years)	mean ± std	60.99 ± 10.88	59.12 ± 10.71	0.528
	range	40–79	38–75	
Gender	Female	5 (22.7%)	13 (39.4%)	0.095
	Male	17 (77.3%)	20 (60.6%)	
Education (years)	mean ± std	16.68 ± 2.51	16.42 ± 2.46	0.53
	range	13–22	11–22	
Handness	Left	2 (9.1%)	5 (15.2%)	0.76
	Right	19 (86.2%)	28 (84.8%)	
	Mixed	1 (4.5%)	0	
Hoehn-Yahr	Stage 0	21 (95.5%)	0 (0.0%)	< 1e-8 *
	Stage1	1 (4.5%)	13 (39.4%)	
	Stage 2	0 (%)	19 (57.6%)	
	Stage 3 ~ 5	0 (0%)	1 (3.0%)	
MoCA	mean ± std	28.18 ± 1.10	27.30 ± 2.48	0.125
	range	27–30	21–30	
Motor onset side	Right/Left		22/11	
Rest Tremor	Yes/No		25/8 (75.8%)	
Ridigity	Yes/No		28/5 (84.8%)	
Bradykinesia	Yes/No/Unkown		31/1/1 (93.9%)	
Posture Instability	Yes/No/Unkown		1/31/1 (3.0%)	
PD Duration	mean ± std		14.58 ± 12.63	
(months)	range		1–49	

### 3.2. Group difference in motor and cognitive function test scores

Table 2 shows the results of group comparison in motor and neuropsychological cognitive test scores. The PD patients had significantly higher MDS-UPDRS Part III motor score than the HC participants (PD: 20.42 ± 10.38; HC: 1.55 ± 2.54; *p* < 1e-10). Martinez-Martin et al. had investigated the relationship between the PD severity and MDS-UPDRS score and suggested that the MDS-UPDRS part III score of 29/30 was the cut-off points of between mild and moderate PD (Martinez-Martin et al., 2015). Thus the PD severity of this patient group was very mild.

**Table 2:** Motor and Cognitive Function Comparison between PD patients and HCs.

Measures	HCs (*N* = 22)				PD patients (*N* = 33)				*p* value
	min	max	mean	Std. err	min	max	mean	std. err^*^	
**MDS-UPDRS III**	0	9	1.55	0.54	7	41	20.42	1.81	< 1e-10^*^
HVLT									
Total Recall	22	70	47.86	2.59	23	80	46.36	2.17	0.104
Delayed Recall	25	80	47.09	3.18	21	80	47.33	2.41	0.064
Retention	26	80	50.23	3.13	21	80	48.12	2.27	0.315
Recog Disc Index	33	80	60.00	3.29	31	80	56.76	2.44	0.677
**JLO Total Score**	9	15	13.27	0.39	9	15	13.21	0.29	0.708
**SDM Total Correct**	28	66	47.14	2.2	24	64	43.39	1.72	0.010^*^
**LNS Total Score**	8	16	11.32	0.46	5	17	10.55	0.47	0.082
SFT									
Animal Score	7	17	10.77	0.51	3	18	11.39	0.6	0.355
Total Score	32	64	48.55	2.1	28	76	50.52	2.1	0.027^*^

*: GLM multivariate analysis with covariates of age, gender, and education, Bonferroni corrected.

For the cognitive function tests, PD patients showed significant worse performance only in the SDM test which evaluates the attention, motor speed, and visual scanning functions (HC: 47.14 ± 10.30; PD: 43.39 ± 9.86; *p* < 0.010). No significant group differences were found in other cognitive function tests including HVLT, JLO, LNST, and SFT. It’s interesting to note that the PD patients in this study showed slightly better executive function than the HC participants in the SFT test (SFT animal score: HC: 10.77 ± 2.39; PD: 11.39 ± 3.45; SFT total score (including animal, fruit and vegetable): HC: 48.55 ± 9,85; PD: 50.52 ± 12.09; corrected *p* < 0.027).

### 3.3. Group difference in motor cerebellar lobular FC

**Figure 2:**
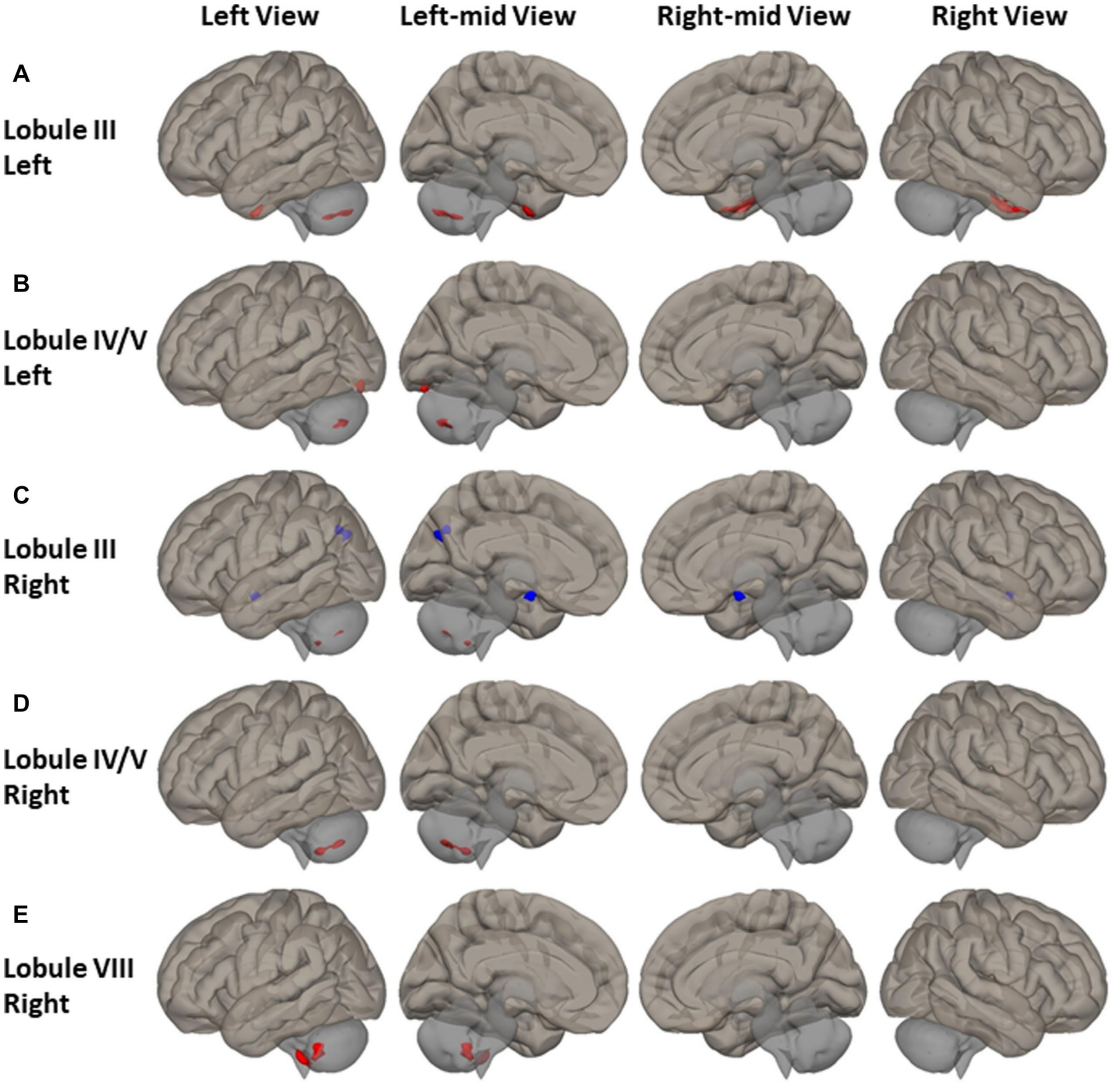
FC difference map between the PD patients and HCs of motor cerebellum seeds of **(A,B)** left lobule III and left lobule IV/V; **(C–E)** right lobule III, right lobule IV/V, and right lobule VIII. Two-sided two sample *t*-test was performed with significant level of voxel uncorrected *p* < 0.008 and cluster FDR-corrected *p* < 0.05. Blue color represents FC of PD less than FC of HCs. Red color represents FC of PD greater than FC of HCs.

Higher FC between motor cerebellum seeds and cortical cortex brain regions as well as within the cerebellum was observed among PD patients (Figure 2 and Supplemental Table S1). Compared to HCs, PD patients had higher FC between left lobule III and bilateral anterior temporal-fusiform cortex, right posterior temporal-fusiform cortex, right inferior temporal gyrus, and right temporal pole, as depicted by red clusters in Figure 2A. PD patients had higher FC between left lobule IV/V and left occipital-fusiform gyrus and inferior lateral occipital cortex, as shown in Figure 2B. PD patients also had higher FC between motor cerebellum seeds and left posterior cerebellum. PD patients demonstrated higher FC between seeds in primary motor cerebellum (left/right lobule III, left/right lobule IV/V) and secondary motor regions located at posterior cerebellum, such as left lobule VIIB and left lobule VIII as shown in Figures 2A–D. FC of right lobule VIII was also found to be higher with left lobule VIII, lobule VII, and Crus II as shown in Figure 2E.

Reduced FC was observed among PD patients between the anterior motor seed of right lobule III and the left cuneal and precuneus cortex, as depicted by the blue clusters in Figure 2C.

### 3.4. Group difference in non-motor cerebellar lobular FC

**Figure 3:**
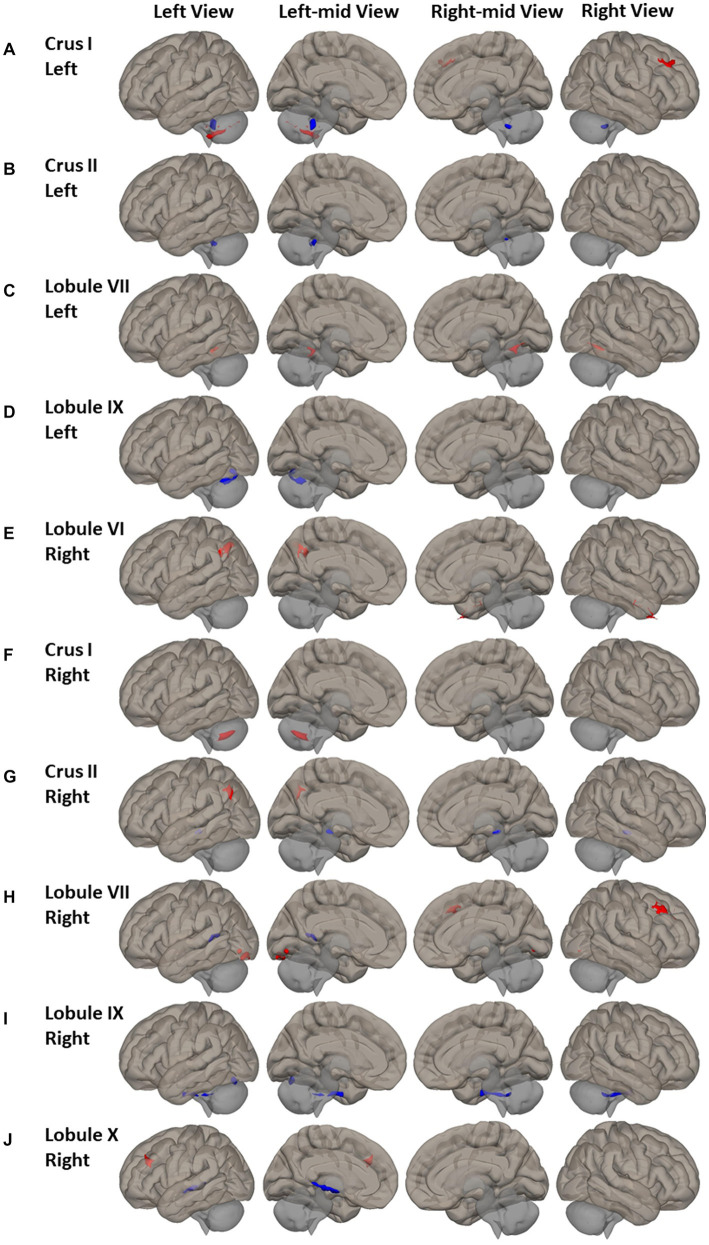
FC difference map between the PD patients and HCs of non-motor cerebellum seeds of **(A–D)** left Crus I, left Crus II, left lobule VII, and left lobule IX; **(E–J)** Right lobule VI, right Crus I, right Crus II, right lobule VII, right lobule IX, and right lobule X. Two-sided two sample t-test was performed with significant level of voxel uncorrected *p* < 0.005 and cluster FDR-corrected *p* < 0.05. Blue color represents FC of PD less than FC of HCs. Red color represents FC of PD greater than FC of HCs.

Wide-spread alterations of FC between non-motor cerebellum seeds and frontal lobe, superior parietal and occipital cortex, and inferior temporal and occipital cortex were identified in PD patients (Figure 3 and Supplemental Table S2). For the seeds of left Crus I and right lobule VII, higher FC was observed in the right middle frontal gyrus and frontal pole of PD patients (Figures 3A,H). The PD had higher FC of right lobule X in the left middle frontal gyrus (Figure 3J). For seeds of right lobule VI and right Crus II, PD showed higher FC in left superior lateral parietal gyrus and angular gyrus (Figures 3E,G). PD also showed higher FC between right lobule VII and bilateral lingual gyrus and left occipital-fusiform gyrus, inferior lateral occipital cortex and occipital cortex. Both the FC of left lobule IX and the FC of right lobule IX in the left occipital-fusiform cortex was found lower in PD patients (Figures 3D,I), which is involved with the higher processing of visual information.

Group differences were also found in FC of non-motor cerebellum seeds to subcortical regions. Compared to HC, PD had lower FC between right Crus II and right lobule IX and brainstem (Figures 3G,I); lower FC of right lobule X with right hippocampus, thalamus, and insular cortex (Figure 3H).

### 3.5. Group difference in cerebellar Vermal FC

**Figure 4:**
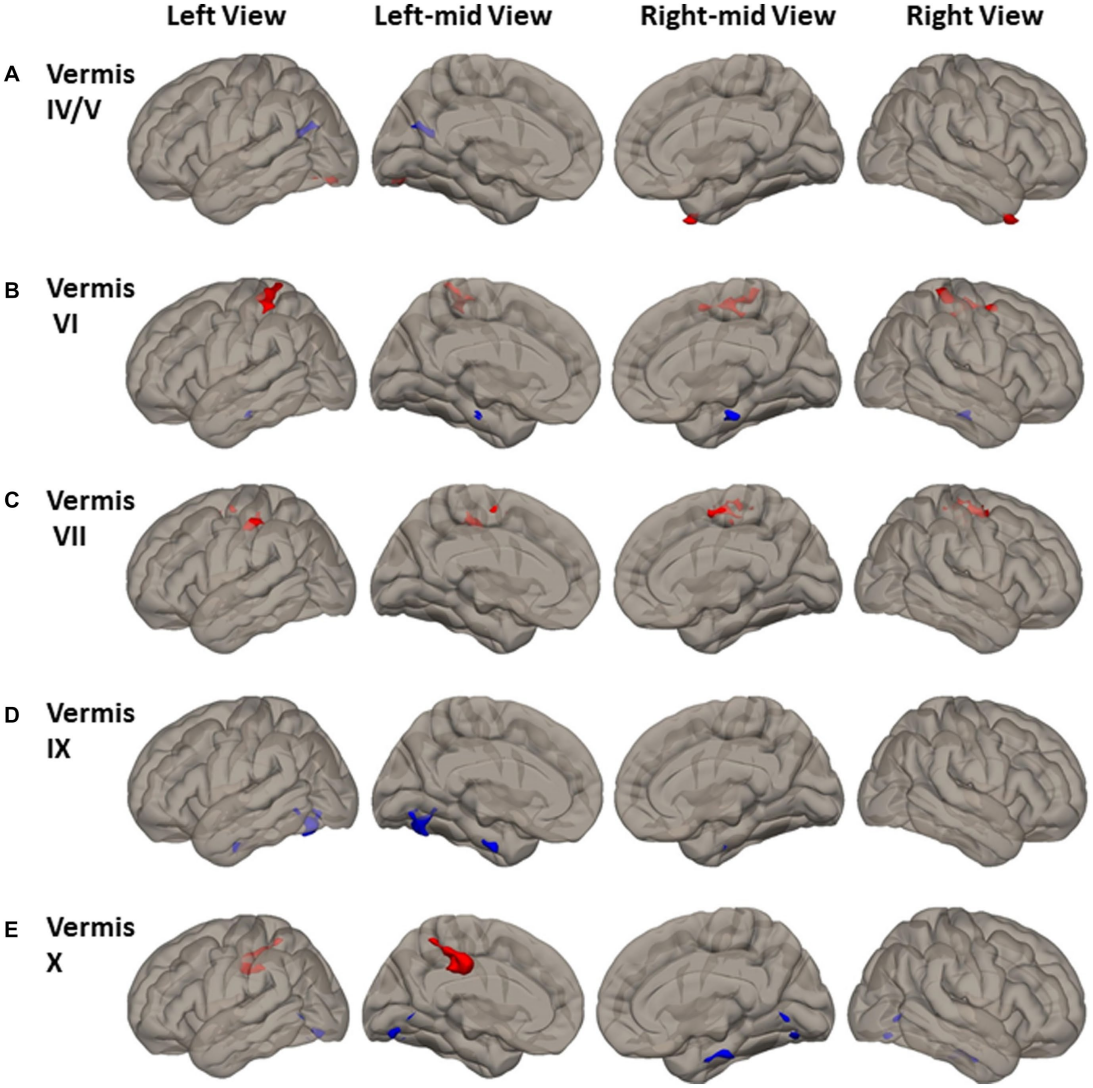
FC difference map between the PD patients and HCs of Vermal cerebellum seeds of **(A)** Vermis IV/V; **(B)** Vermis VI; **(C)** Vermis VII; **(D)** Vermis IX; and **(E)** Vermis X. Two-sided two sample t-test was performed with significant level of voxel uncorrected *p* < 0.005 and cluster FDR-corrected *p* < 0.05. Blue color represents FC of PD less than FC of HCs. Red color represents FC of PD greater than FC of HCs.

Altered cerebellar vermal FC was observed in cerebral cortical cortex (Figure 4 and Supplemental Table S3). Compared to HCs, PD had higher FC between vermis IV/V and left inferior lateral occipital cortex, occipital-fusiform, and the right temporal pole (Figure 4A). PD had higher FC of vermis VI, VII, and X to the somatomotor cortical regions. Specifically, higher FC was found between vermis VI and bilateral precentral gyrus, postcentral gyrus, superior parietal lobule, and left superior marginal gyrus (Figure 4B); between vermis VII and bilateral supplemental motor area, precentral gyrus, postcentral gyrus, left supramarginal gyrus, and right superior parietal gyrus (Figure 4C); between vermis X and left postcentral gyrus, superior parietal lobule, and anterior supramarginal gyrus (Figure 4E).

Reduced FC in PD was found between vermis IV/V and visual and default mode network (DMN) region of precuneus and left cuneal cortex (Figure 4A). Lower FC in PD was found between both the vermis IX and vermis X and visual processing cortical regions of left lingual gyrus and occipital-fusiform gyrus (Figures 4D,E). Lower FC in PD was also found between vermis VI and vermis X and brainstem (Figures 4B,E).

### 3.6. Association between MDS-UPDRS III score and cerebellar FC

**Figure 5:**
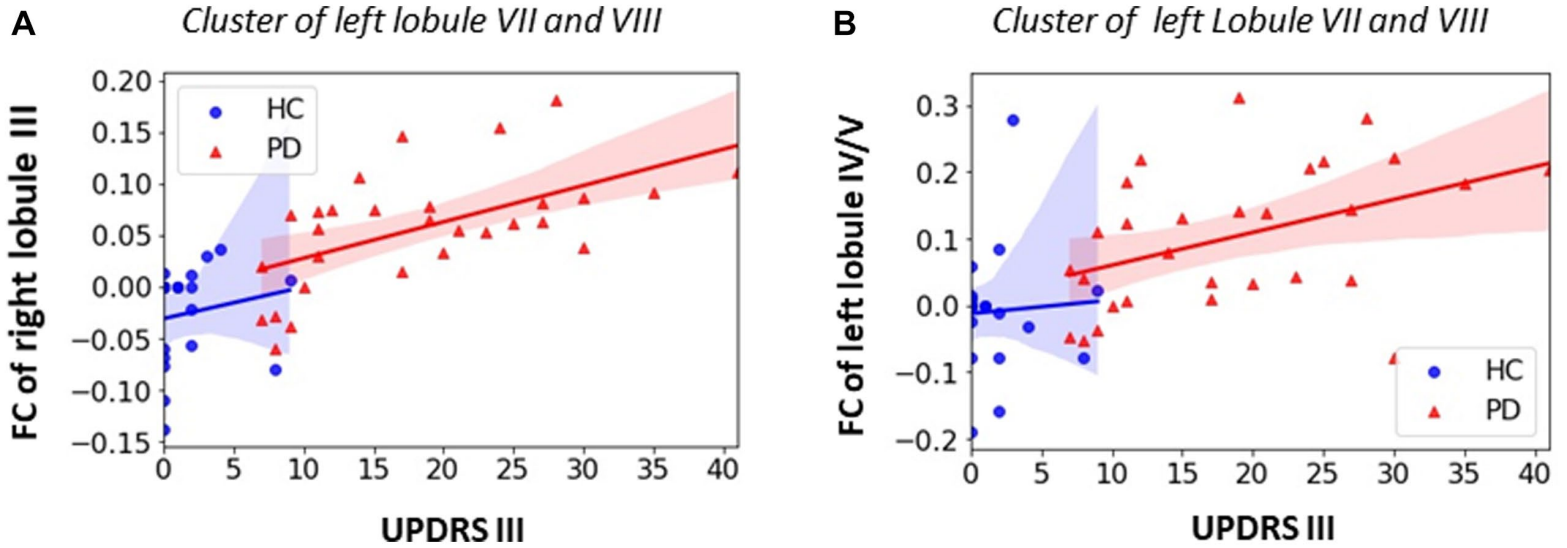
Association between FC within significant clusters and UPDRS motor score in PD group. **(A)** Positive correlation for FC of right lobule III in left lobule VII/VIII (PD: *r* = 0.603, *p* = 0.001); **(B)** positive correlation for left lobule IV/V in left lobule VII/VIII (*r* = 0.449, *p* = 0.022). All the enhanced motor cerebellar FC are associated with worse motor performance. Non-parametric correlation with controlling covariates of age, sex and education was utilized and significant level was defined as *p* < 0.05.

Significant positive correlations between MDS-UPDRS III score and the FC of primary motor cerebellar lobules were observed in PD patients. The PD patients showed that higher MDS-UPDRS III motor scores (i.e., higher motor dysfunction or worse motor function) were associated with increased FC between right lobule III and secondary motor cerebellar lobules of left lobule VIIB and lobule VIII (r = 0.553, *p* = 0.003, Figure 5A); and increased FC between left lobule VI/V and left lobule VIIB and lobule VIII (r = 0.408, *p* = 0.038, Figure 5B).

### 3.7. Association between cognitive function test scores and cerebellar FC

**Figure 6:**
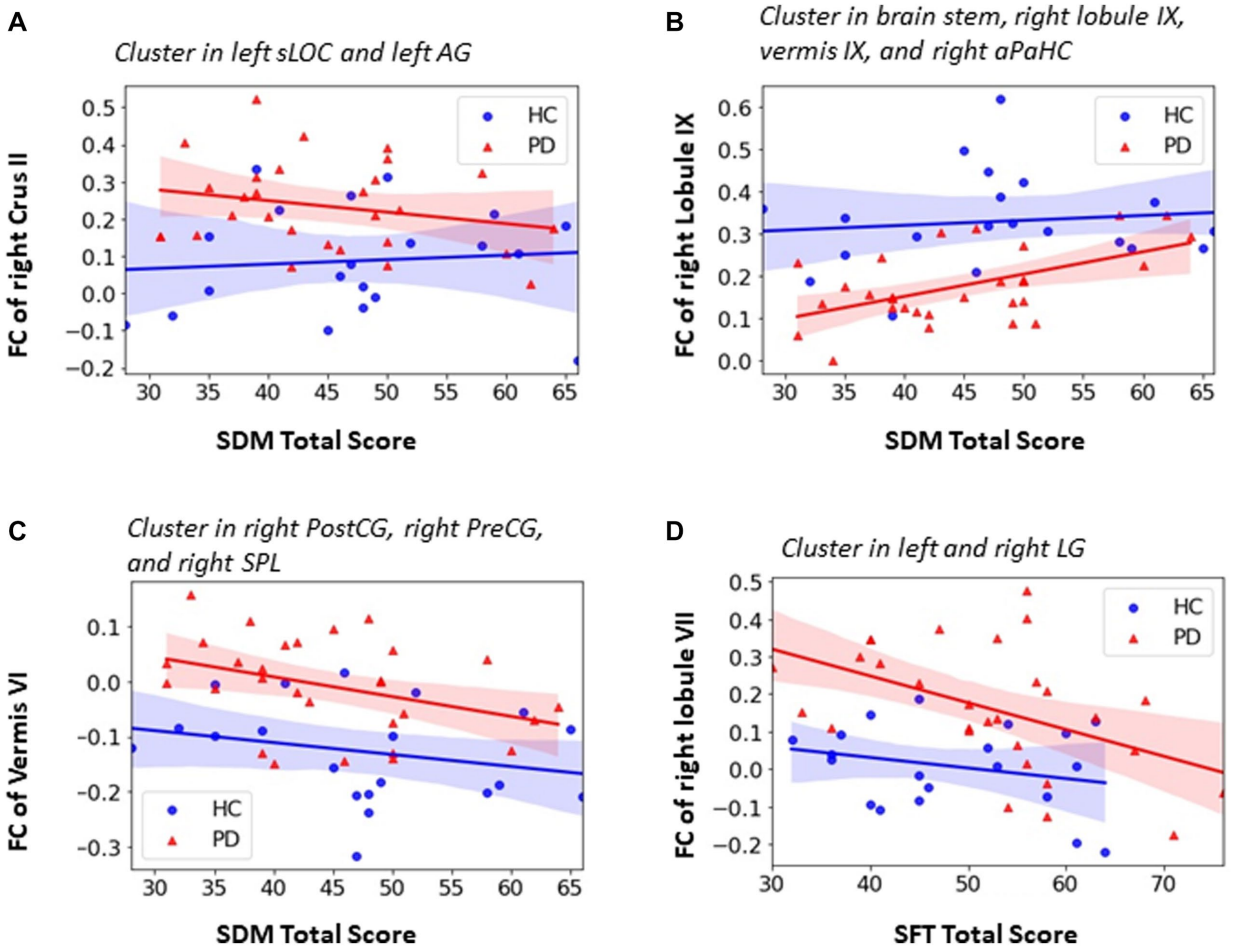
Association between FC within significant clusters and neurophysiological test score in PD group. **(A)** negative correlation between SDM total score and the FC of right Crus II in left sLOC and left AG (PD: *r* = −0.395, *p* = 0.046); **(B)** positive correlation between SDM total score and the FC of right lobule IX in brainstem, right aPaHC, right lobule IX and vermis IX (*r* = 0.455, *p* = 0.019); **(C)** negative correlation between SDM total score and the FC of vermis VI in right PostCG, right PreCG and right SPL (*r* = −0.432, *r* = 0,027); **(D)** negative correlation between SFT total score and the FC of lobule VII in bilateral LG (PD: *r* = −0.406, *p* = 0.039). Partial correlation with controlling of age, sex and education was utilized and significant level was defined as *p* < 0.05.

Among all the cognitive function tests, only SDM and SFT test scores showed significant difference between the PD and HC groups (as shown in Table 2). Thus, further partial correlation between the SDM and SFT test scores and the cerebellar FC was performed controlling for age, sex, and education and the results were shown in Figure 6.

Both significant positive and negative correlation were observed between the cerebellar FC and SDM test scores for attention, perceptual speed, motor speed, and visual scanning. As shown in Figure 6A, the PD patients had higher FC of right Crus II in the left superior lateral occipital cortex (sLOC) and angular gyrus (AG) which belong to the visual and attention network. The FC of right Crus II was negatively correlated with SDM total score in PD group (r = −0.395, *p* = 0.046) whereas the trend was opposite in the HC group although not significant (r = 0.185, *p* = 0.477) (Figure 6A). As shown in Figure 6C, the PD patients showed higher FC between vermis VI and right PreCG, PostCG and SPL which belong to sensori-motor network and were negatively correlated with SDM total score in both PD group (*r* = −0.432, *p* = 0.027) and HC group (*r* = −0.428, *p* = 0.088) (Figure 6C). Significant positive correlation was found between the SDM total score and the FC of right lobule IX in subcortical and its neighboring cerebellar regions including brainstem, right anterior parahippocampal gyrus, right lobule IX, and vermis IX in PD patients group (*r* = 0.455, *p* = 0.019) (Figure 6B).

As shown in Figure 6D, PD patients showed higher FC of right lobule VII in the left and right lingual gyrus (LG) when compared with HCs. The lingual gyrus is involved in processing of visual information. The PD patients also showed significant negative correlation between the FC of right lobule VII and SFT total score (r = −0.406, *p* = 0.039), indicating that higher FC of right lobule VII in left and right LG was associated with poor executive function.

## 4. Discussion

The present study aimed to investigate the alteration of cerebellar functional connectivity (FC) patterns among early-stage, drug-naïve PD patients and its relationship to early changes in motor and cognitive function. The existing literature lacks sufficient evidence regarding such alterations in this specific patient population, as most studies have focused on moderate to advanced PD patients receiving treatment. Consequently, it remains unclear whether the reported FC changes are primarily due to the disease itself, the medication used, or a combination of both factors. This study is aimed to address this paucity and our findings revealed several key results. Firstly, we observed increased intra-cerebellar FC between the first and second representations of the motor cerebellum. Additionally, motor-cerebellar FC was enhanced in the ventral visual pathway, specifically in the inferior temporal gyrus and inferior lateral occipital gyrus, while reduced FC was observed in the cuneus and dorsal posterior precuneus within the dorsal visual pathway. Furthermore, we identified increased non-motor cerebellar FC in cortical areas associated with attention, language, and visual networks. Moreover, vermal FC was found to be increased within the somatomotor cortical network. Conversely, we observed decreased FC between non-motor and vermal subdivisions with the brainstem, thalamus, and hippocampus. Furthermore, our association analysis revealed significant correlations between cerebellar FC and clinical measures. Specifically, enhanced FC within the motor cerebellum was positively associated with the Unified Parkinson’s Disease Rating Scale (UPDRS) motor score, while increased non-motor and vermal FC showed negative associations with cognitive function test scores, such as the Symbol Digit Modalities Test (SDM). Overall, our findings provide evidence that cerebellar FC changes are involved in the pathophysiology of PD and highlight the potential of cerebellar FC as a biomarker for early PD diagnosis and monitoring.

Motor Cerebellar FC Changes in early stage drug-naïve PD patients.

Previous work detailing the general pattern of cerebellar FC has already revealed that there are two somatomotor representations in the cerebellum that are primarily involved in motor processing (Stoodley and Schmahmann, 2010; Bernard et al., 2012, Guell et al., 2018a,b). The first somatomotor representation is located at the anterior part of the cerebellum and encompass the lobule I-III, IV and V. The second somatomotor representation is located at the posterior part of the cerebellum and includes the lobule VIII. Previous studies showed that the M1 had FC with both the first representation and second representation although they are anatomically distant (Bernard et al., 2012; Guell et al., 2018b). In this study, we found increased intra-cerebellar FC consistent with previous studies (Cerasa et al., 2016; Tuovinen et al., 2018; Gratton et al., 2019; Yoo et al., 2019; Kaut et al., 2020). Specifically, we found out that enhanced FC between all the subdivisions of the first somatomotor representation (left and right lobule III, left and right lobule IV/V) and the second somatomotor representation on the left side (left lobule VIIb/VIII) in PD patients when compared with HCs. In general, increased levels of cerebellar connectivity were most commonly related with improved performance, supporting the compensatory cerebellum involvement in PD (Wu and Hallett, 2013; Palmer et al., 2020). However, in this study we found out that greater FC between right lobule III and left lobule VIIb/VIII and greater FC between left lobule IV/V and left lobule VIIb/VIII are positively correlated with UPDRS motor score, indicating that greater FC was associated with worse motor performance in the early stage drug-naïve PD patients. To interpret this phenomenon, Festini et al. cited a plausible explanation that at a certain level, reliance on the cerebellum is no longer feasible to compensate for other dysfunctional neural systems (i.e., the striatum), and cerebellar recruitment becomes affiliated with impaired behavior which also been used in healthy cognitive aging, regarding compensatory frontal recruitment (Festini et al., 2015). Increased FC between the motor representations and cortical regions were also found in this study. Unlike previous studies which showed enhanced FC between cerebellar motor lobules or cerebellar sensorimotor network (SMN) and the cortical somatomotor regions in PD patients (Tuovinen et al., 2018; Palmer et al., 2020), we did not obverse significant motor cerebellar FC changes in the somatomotor cortical regions in the early stage drug-native PD patients. Instead, increased motor cerebellar FC was observed in the left and right inferior temporal lobe (including right inferior temporal gyrus, right temporo-fusiform cortex, right temporal pole, left temopro-fusiform cortex, left occipito-fusiform gyrus) and left inferior lateral occipital cortex (iLOC) which is located in the ventral visual stream involving the higher processing of visual information for the purpose of visual perception (Sheth and Young, 2016). Visuomotor impairment is often found in PD patients (Chen et al., 2016), and it is associated with basal ganglia dysfunction and the reduced function of other motor brain regions that connect to basal ganglia such as motor cortex and cerebellum (Wu and Hallett, 2013). Our finding may indicate that the cerebellum may be involved in the visuomotor control and the enhanced FC between the motor cerebellum and ventral visual pathway may suggest the compensatory effect of the cerebellum to maintain the visuomotor function in the early stage drug-naïve PD patients.

Reduced FC was found between one of the subdivisions of motor cerebellum (right lobule III) and left cuneus and dorsal posterior precuneus (dorsal visual stream), which is involved in basic visual processing (Cavanna and Trimble, 2006; Zhang and Li, 2012; Parker et al., 2014). This reduced FC may be an early sign of pathophysiological changes associated with visuomotor function impairment in the early stage drug-naïve PD patients.

### 4.1. Non-motor cerebellar FC changes in early stage drug-naïve PD patient

Resting-state and task fMRI studies have shown that cerebellum is activated in relation to non-motor functions, such as language, spatial processing, working memory, executive function, and emotional processing, and revealed a triple non-motor representations (first = VI/Crus I, second = Crus II/VIIb, third = IX/X) along with detailed topological functional gradient mapping of the cerebellum (Stoodley and Schmahmann, 2010; Bernard et al., 2012; Guell et al., 2018a). Meanwhile, many studies have shown that cerebellum, together with the basal ganglia, is involved in both motor and non-motor functions in PD (Solstrand Dahlberg et al., 2020). Consistent with previous studies, our results demonstrated wide-spread increased FC between subdivisions of non-motor cerebellum and their corresponding functional cortical regions covering attention network, language network, and visual network. For example, we observed that left Crus I and right lobule VII showed greater FC in right middle frontal gyrus and right frontal pole which is the convergence cortical area of the ventral attention network and dorsal attention network. This is consistent with previous resting-state fMRI studies which revealed that Crus I is cerebellum representation of dorsal attention network and lobule VII is the cerebellum representation of ventral attention network. Based on task fMRI imaging studies, the language processing task activated the lobule VI and Crus I/II (Guell et al., 2018a) and a cerebellar-occipital-fusiform-thalamic network centered around bilateral lingual gyrus for word association was identified (Ghosh et al., 2010). Our results showed that right lobule VI and right Crus II had greater FC in left superior lateral occipital cortex (sLOC) and left angular gyrus (AG) which is involved in object recognition and higher language processing. We also found enhanced FC of right lobule VII with left primary visual cortex (left inferior lateral occipital cortex and occipital pole) and left ventral visual stream (left occipito-fusiform gyrus, and bilateral lingual gyrus). Especially, the FC of right lobule VII with bilateral lingual gyrus is significantly negatively associated with sematic fluency test (SFT) score, which indicates that enhanced FC is associated with better executive function for word associated task. The FC of right Crus II is significantly negatively associated with SDM test score for the integrated function of attention, motor speed, and visual scanning. Taken together these findings demonstrate that the changed non-motor cerebellar FC may contribute to the cognitive function impairment in early stage drug-naïve PD patients.

### 4.2. Greater Vermal FC in sensorimotor cortical network

In this study, we intentionally separated the vermis from the hemispherical lobules of the cerebellum and investigated whether the vermis had unique role in PD. The vermis, as part of spinocerebellum, classically is thought to receive somatic sensory, visual, and auditory inputs *via* ascending spinal pathways and has intimate connections with other parts of cerebellum (Kandel et al., 2000). Recently, the cerebellar vermis was found to have projections from motor areas of cerebral cortex (Coffman et al., 2011). In this study, the most pronounced greater FC from vermis subdivisions (Vermis VI, VII, and X) was found in the sensorimotor cortical areas including the bilateral primary motor cortex (PreCG), bilateral primary somatosensory cortex (PostCG), bilateral supplemental motor cortex (SMA), and left supramarginal gyrus. It’s noted that only the vermis, not the hemispherical lobular subdivisions, had significantly greater FC with sensorimotor cortical network and the resulting greater FC from the vermal FC which shifts from being negative in HCs to more positive in the early stage drug-naïve PD patients. Similarly, Maiti et al., revealed that increased vermal FC (using entire vermis as a seed) with the sensorimotor cortices, in the absence of relevant vermal or cortical atrophy, may predict future gait impairment, one of the motor symptoms in PD (Maiti et al., 2021). Furthermore, our association analysis shows that, in early stage drug-naïve PD patients, the FC between vermis VI and right PreCG and right PostCG was significantly negatively correlated with the Symbol-Digit Modalities test (SDM) which involves an integrated function of attention, perceptual speed, motor speed, and visual scanning (Figure 6C). The greater FC is associated with worse performance in SDM test which might be an indicator of pathophysiological process of cognitive and motor deficit in PD patients.

### 4.3. Decreased lobular FC and vermal FC in brainstem

Another one of the intriguing findings in this study was the decreased FC between several subdivisions (right Crus II, right lobule IX, vermis VII, vermis IX, and vermis X) of the cerebellum and the brainstem (Figures 3, 4). Previous post-mortem studies showed that brainstem plays an important role in PD by revealing that pathological changes in PD first appear primarily in the brainstem with loss of serotonergic and cholinergic neurons and with subsequent progression to substantia nigra pars compacta with prominent loss of dopaminergic neurons (Braak et al., 2004; Hacker et al., 2012; Herrington et al., 2017). A resting-state fMRI FC study of the striatum demonstrated reduced FC between the striatum and extended brainstem reinforcing the importance of the brainstem in the pathophysiology of PD (Hacker et al., 2012). Our findings of reduced FC between cerebellum and brainstem, together with reduced FC between cerebellum and thalamus and hippocampus (Figures 3H,J), further supports the idea that both the cerebellum and brainstem contribute to the pathophysiology process of PD.

Altogether, our results demonstrated both increased and reduced cerebellar FC in the early stage drug-naïve PD patients when compared with HCs and their association with the motor and non-motor function scores further support the idea that the cerebellum plays an important role in PD even at early stage.

### 4.4. Limitations

PPMI database has about one hundred of robust and reliable longitudinal resting-state fMRI data of PD patients at the time of writing this manuscript. However, the sample size for newly diagnosed drug-naïve PD patients is still limited. In order to include more drug-naïve PD patients, we obtained the rs-fMRI and behavioral data not only at baseline but also from other time points when no PD-related medication treatment was yet administrated. Thus even though 70% of the early stage PD patients were newly diagnosed with PD duration less than 6 months, the PD duration at the chosen visit of rs-fMRI scan was extended to 33 months. As the number of participants increases for the PPMI study it would be of interest to see if the findings from this study still hold on a more homogenous data from initial diagnosis. Future studies should focus on the long-term status of cerebellar connectivity changes as the disease progresses.

Another limitation is that we used the anatomic parcellation of the cerebellum according to the AAL atlas (Diedrichsen et al., 2009) which may not well reflect the functional segregation of the cerebellum. For example, cerebellar lobule V is involved in both motor and cognitive function. Future studies may be conducted in a functionally defined cerebellar atlas (Guell et al., 2018b; King et al., 2019).

Furthermore, it is important to acknowledge that our analysis examining the association between functional connectivity (FC) and behavior did not account for two significant factors: PD disease duration and cortical/cerebellum atrophy, both of which are known to be important considerations. Disease duration was not included as a covariate in our analysis because it was not found to be correlated with MDS-UPDRS III and cognitive scores in these early-stage, drug-naïve PD patients. Additionally, cortical/cerebellum atrophy was not included as a controlling covariate because we already included age as a covariate, which is known to be associated with cortical/cerebellar atrophy. But this might be a potential limitation of this study.

## 5. Conclusion

Cerebellar FC in early stage drug-naïve PD patients has not received much attention. We have demonstrated multiple changes of FC including those related to motor and cognition within the cerebellum and various brain cortical/subcortical regions in early stage drug-naïve PD patients. Our findings, while reflective of early symptoms of PD, also suggest a possible compensatory mechanism to maintain the visuomotor function. More studies are required to confirm our findings and to investigate the pathophysiological changes in cerebellar connectivity in PD.

## Data availability statement

The raw data supporting the conclusions of this article will be made available by the authors, without undue reservation.

## Ethics statement

The studies involving human participants were reviewed and approved by Data used in the preparation of the manuscript were obtained from the Parkinson’s Progression Markers Initiative (PPMI) database (www.ppmi-info.org/data). PPMI – a public-private partnership – is funded by the Michael J. Fox Foundation for Parkinson’s Research Funding Partners. The patients/participants provided their written informed consent to participate in this study.

## Author contributions

LJ: conceptualization, methodology, formal analysis, investigation, writing-original draft, and writing review and editing. JZ: methodology and writing review and editing. AF and PF: writing-reviewing and editing. RG: conceptualization, methodology, supervision, and writing reviewing and editing. All authors contributed to the article and approved the submitted version.

## Conflict of interest

The authors declare that the research was conducted in the absence of any commercial or financial relationships that could be construed as a potential conflict of interest.

## Publisher’s note

All claims expressed in this article are solely those of the authors and do not necessarily represent those of their affiliated organizations, or those of the publisher, the editors and the reviewers. Any product that may be evaluated in this article, or claim that may be made by its manufacturer, is not guaranteed or endorsed by the publisher.

## Footnotes

^1^www.ppmi-info.org/data
