# Supplementary material for: Cerebellar functional connectivity change is associated with motor and neuropsychological function in early stage drug-naïve patients with Parkinson’s disease
Source: Front Neurosci. 2023 Jun 22;17:1113889. doi: 10.3389/fnins.2023.1113889 (PMC10324581; doi:10.3389/fnins.2023.1113889)
Supplement: Supplementary file 1 [file Data_Sheet_1.PDF]

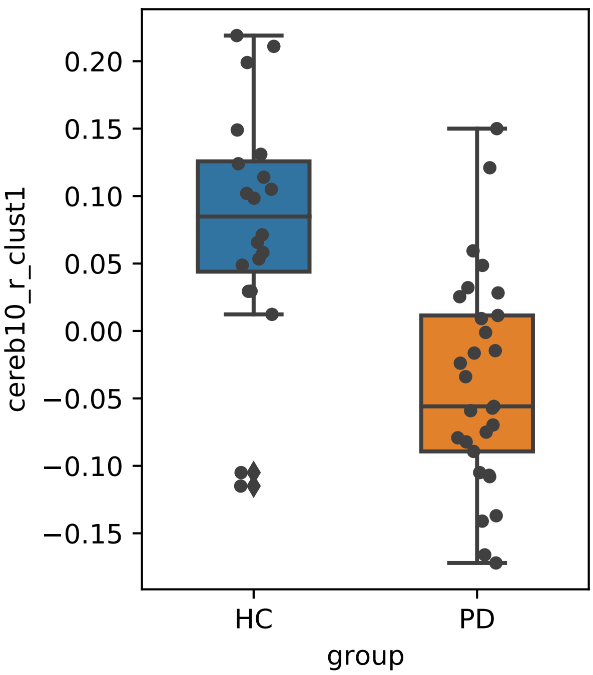

0.20
0.15
0.10
0.05
0.00
−0.05
−0.10
−0.15
cereb10_r_clust1
HC
PD
group

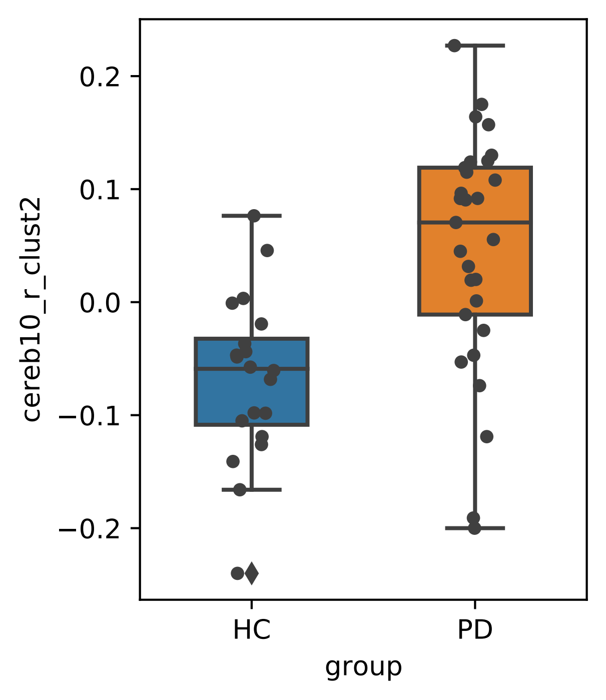
0.2
0.1
0.0
−0.1
−0.2
cereb10_r_clust2
HC
PD
group

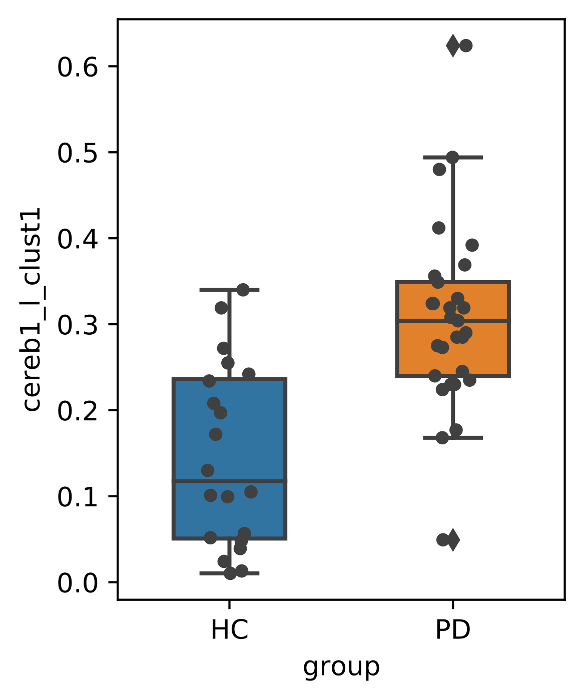

0.0
0.1
0.2
0.3
0.4
0.5
0.6
cereb1_l_clust1
HC
PD
group

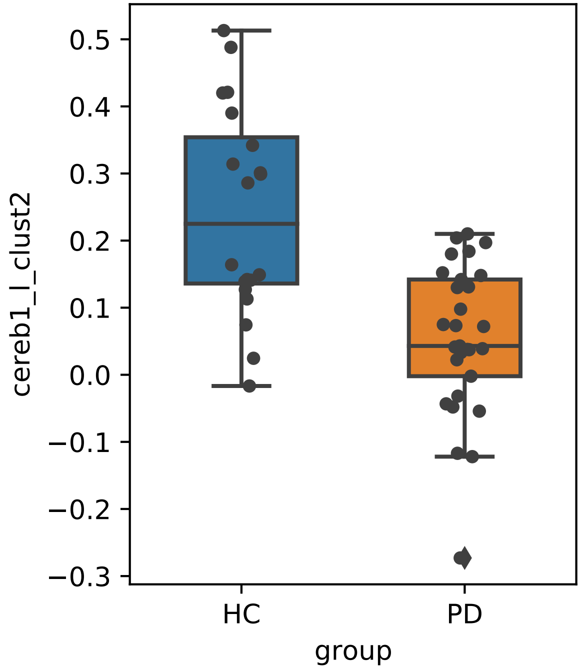

0.5
0.4
0.3
0.2
0.1
0.0
−0.1
−0.2
−0.3
cereb1_l_clust2
HC
PD
group

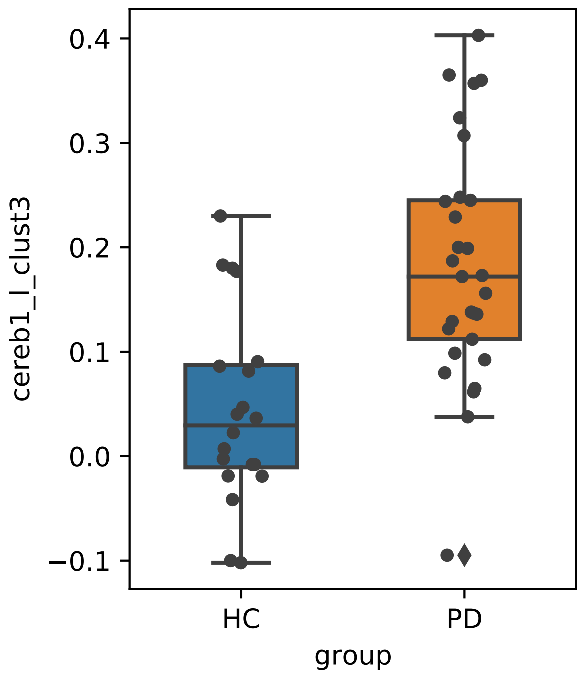

cereb1_l_clust3
0.4
0.3
0.2
0.1
0.0
−0.1
HC
PD
group

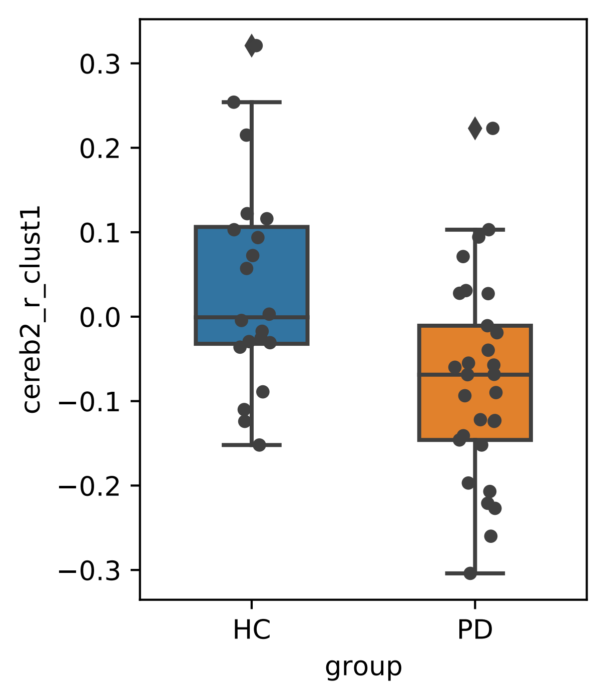
0.3
0.2
0.1
0.0
−0.1
−0.2
−0.3
cereb2_r_clust1
HC
PD
group

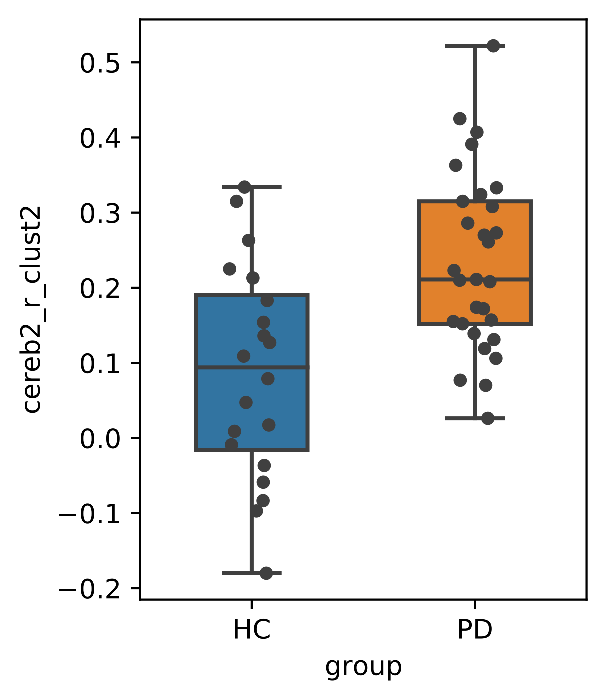

0.5
0.4
0.3
0.2
0.1
0.0
−0.1
−0.2
cereb2_r_clust2
HC
PD
group

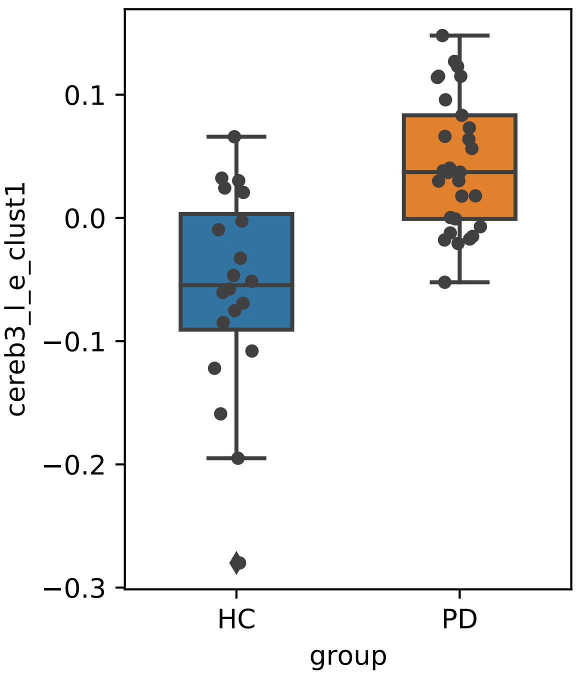

cereb3_l_e_clust1
0.1
0.0
−0.1
−0.2
−0.3
HC
PD
group

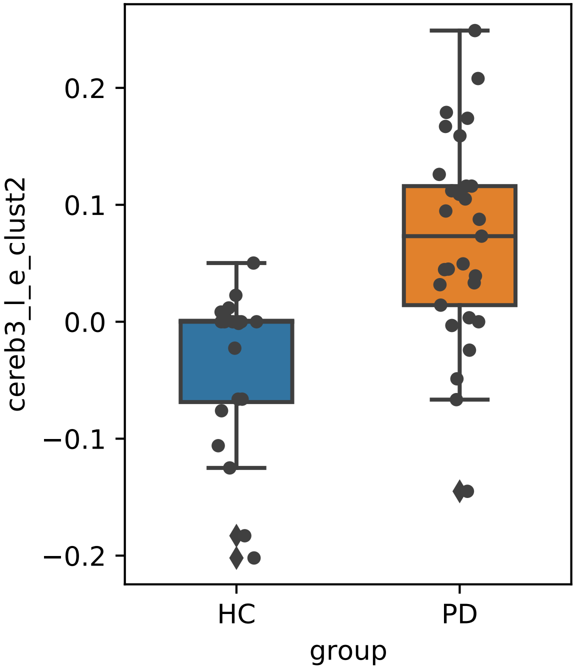

cereb3_l_e_clust2
0.2
0.1
0.0
−0.1
−0.2
HC
PD
group

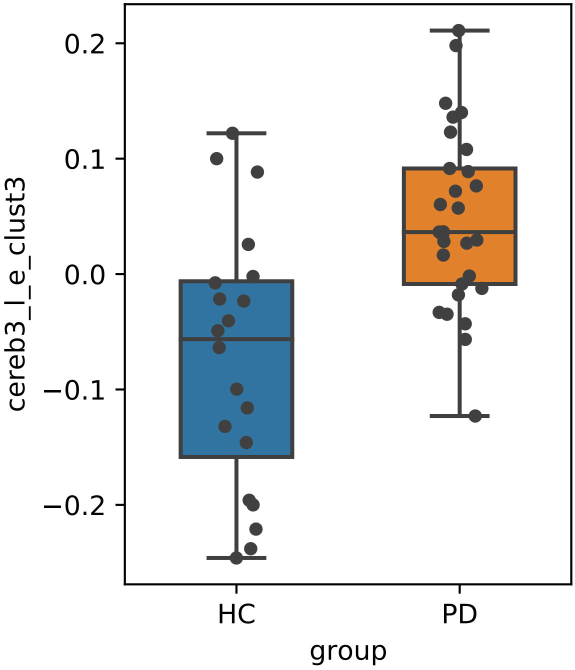

0.2
0.1
0.0
−0.1
−0.2
cereb3_l_e_clust3
HC
PD
group

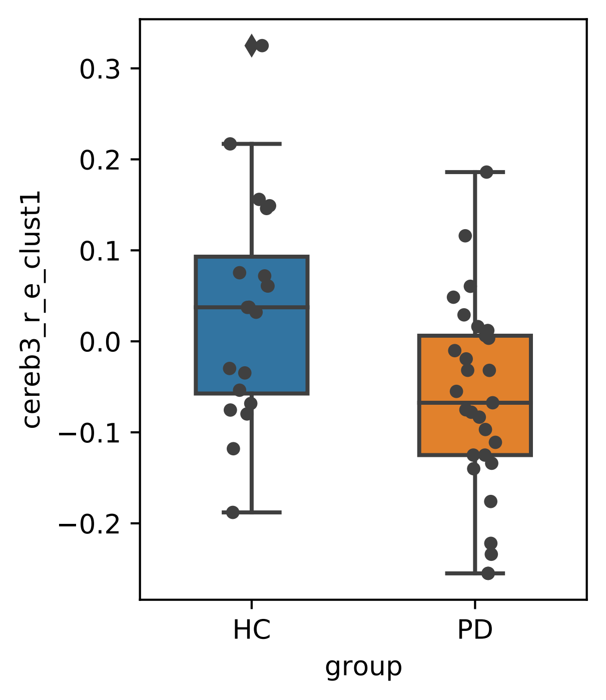

0.3
0.2
0.1
0.0
−0.1
−0.2
cereb3_r_e_clust1
HC
PD
group

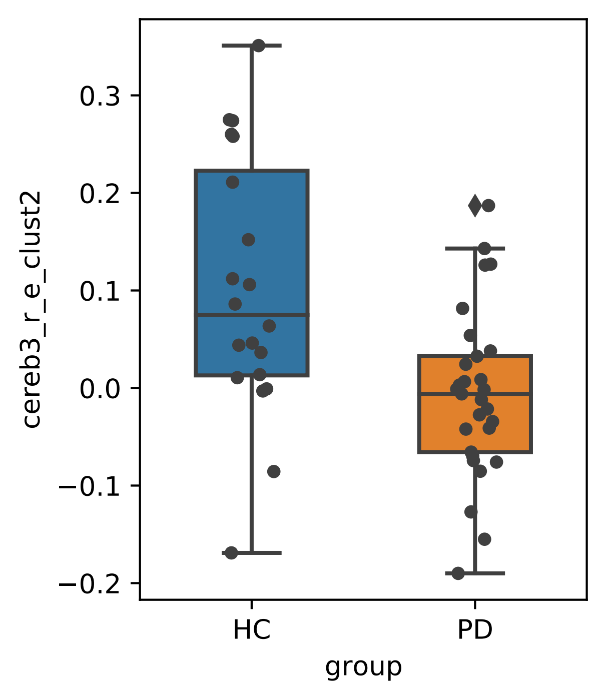

0.3
0.2
0.1
0.0
−0.1
−0.2
cereb3_r_e_clust2
HC
PD
group

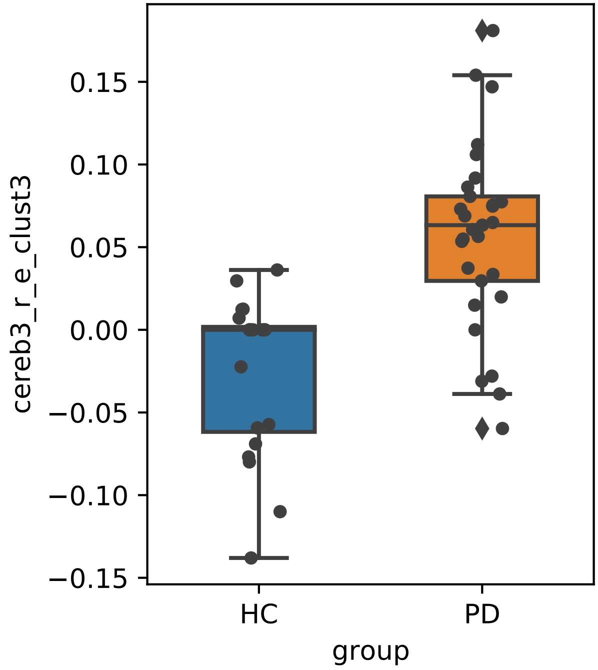
0.15
0.10
0.05
0.00
−0.05
−0.10
−0.15
cereb3_r_e_clust3
HC
PD
group

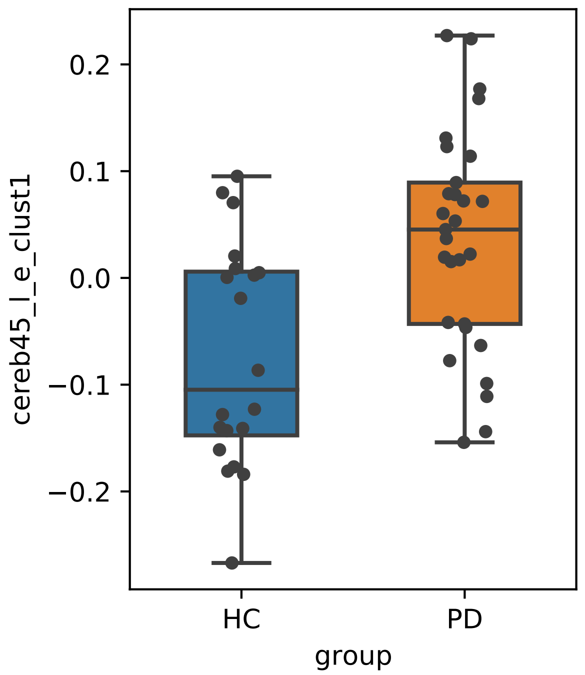

0.2
0.1
0.0
−0.1
−0.2
cereb45_l_e_clust1
HC
PD
group

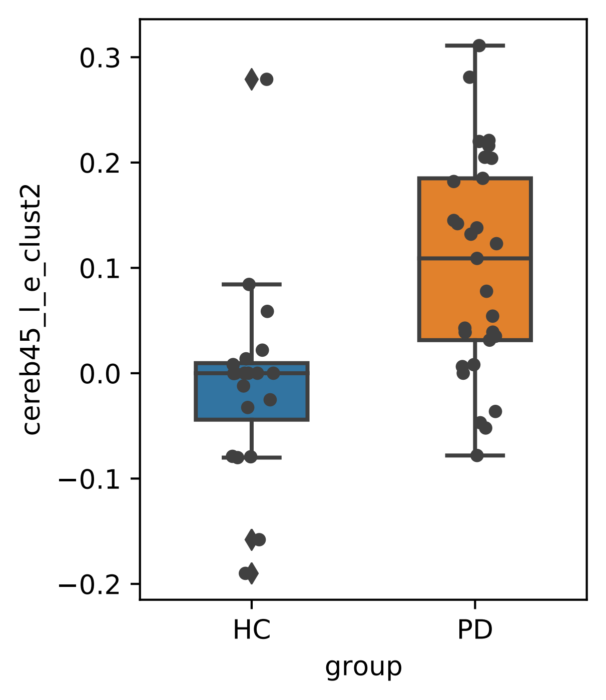

0.3
0.2
0.1
0.0
−0.1
−0.2
cereb45_l_e_clust2
HC
PD
group

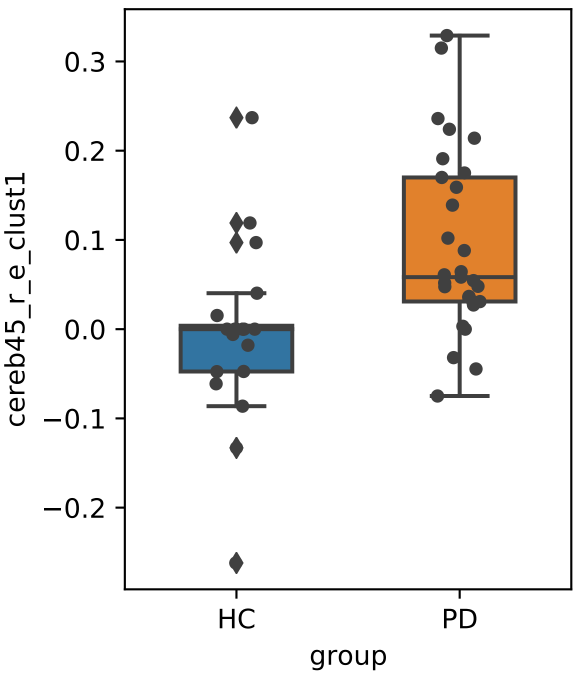

0.3
0.2
0.1
0.0
−0.1
−0.2
cereb45_r_e_clust1
HC
PD
group

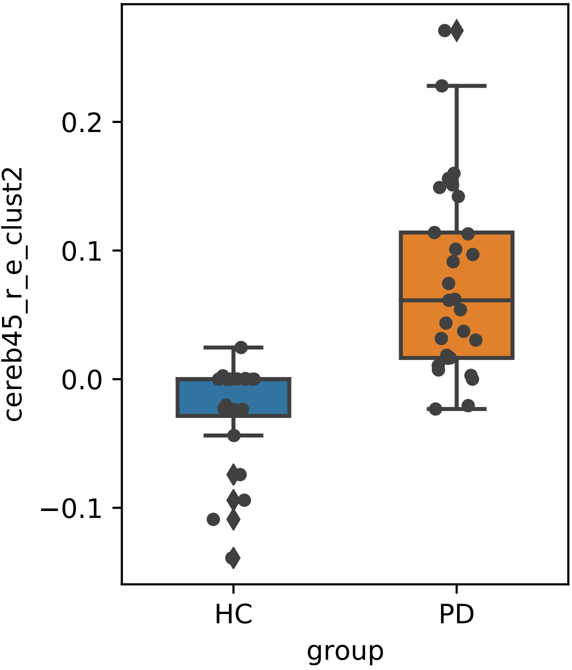

cereb45_r_e_clust2
0.2
0.1
0.0
−0.1
HC
PD
group

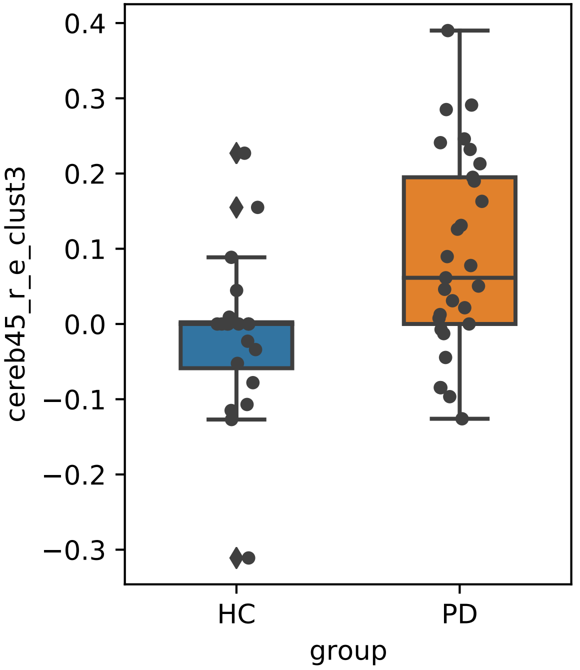

0.4
0.3
0.2
0.1
0.0
−0.1
−0.2
−0.3
cereb45_r_e_clust3
HC
PD
group

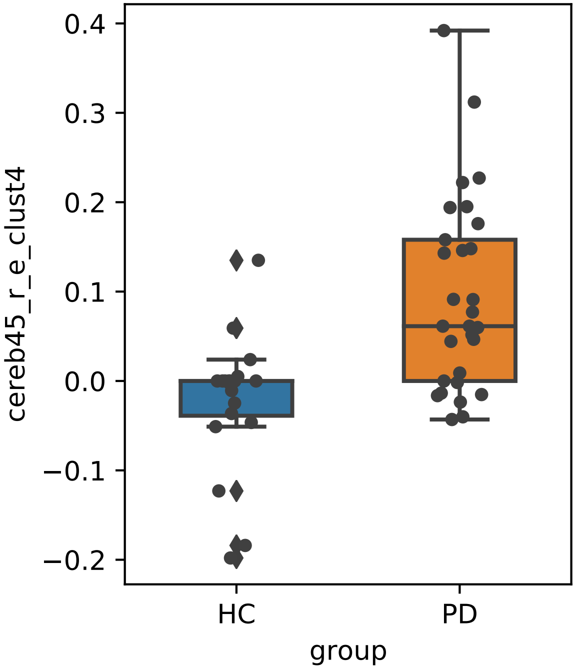

0.4
0.3
0.2
0.1
0.0
−0.1
−0.2
cereb45_r_e_clust4
HC
PD
group

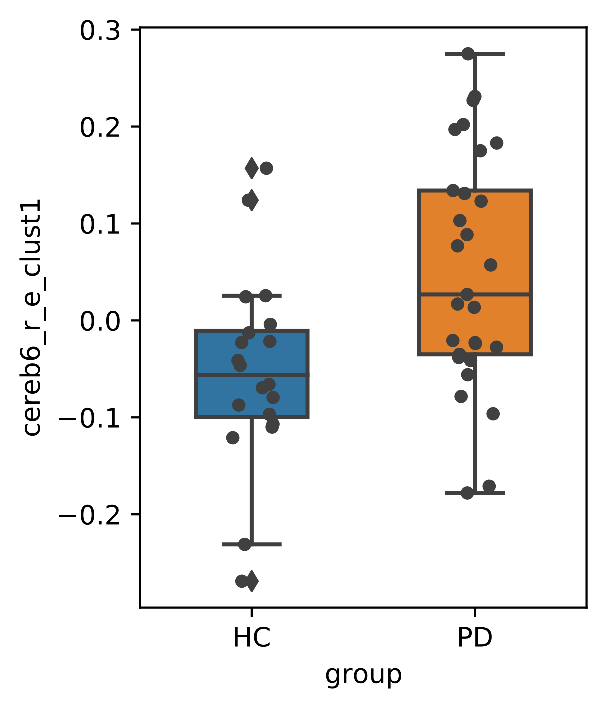

0.3
0.2
0.1
0.0
−0.1
−0.2
cereb6_r_e_clust1
HC
PD
group

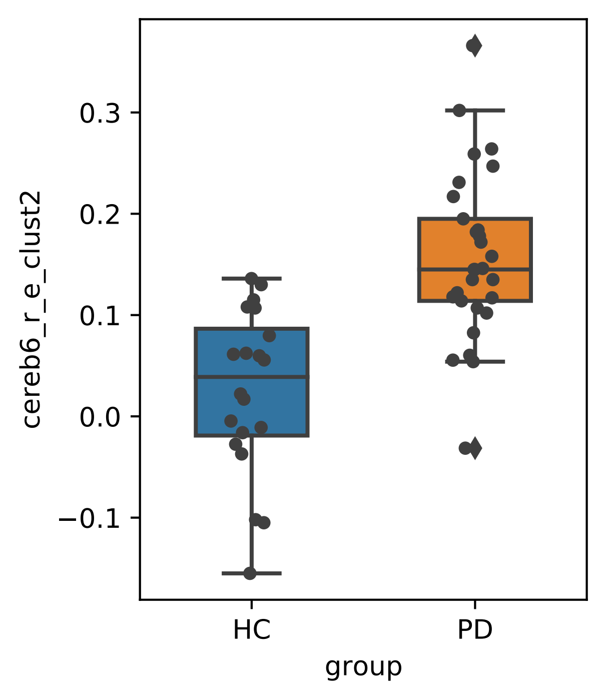
0.3
0.2
0.1
0.0
−0.1
cereb6_r_e_clust2
HC
PD
group

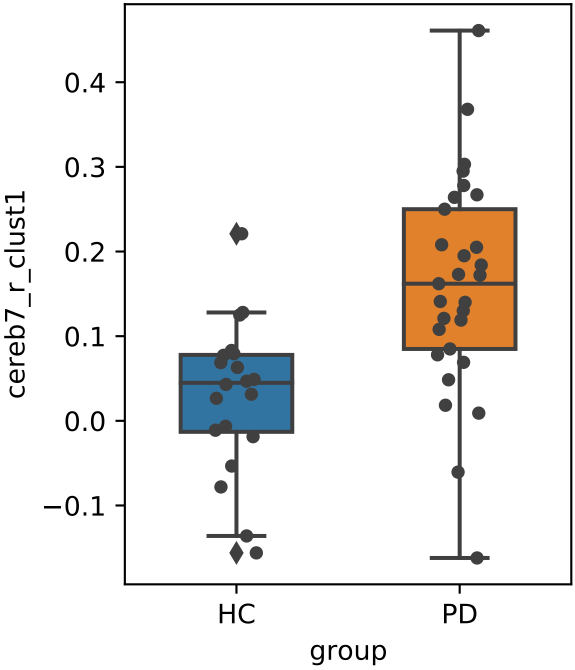

0.4
0.3
0.2
0.1
0.0
−0.1
cereb7_r_clust1
HC
PD
group

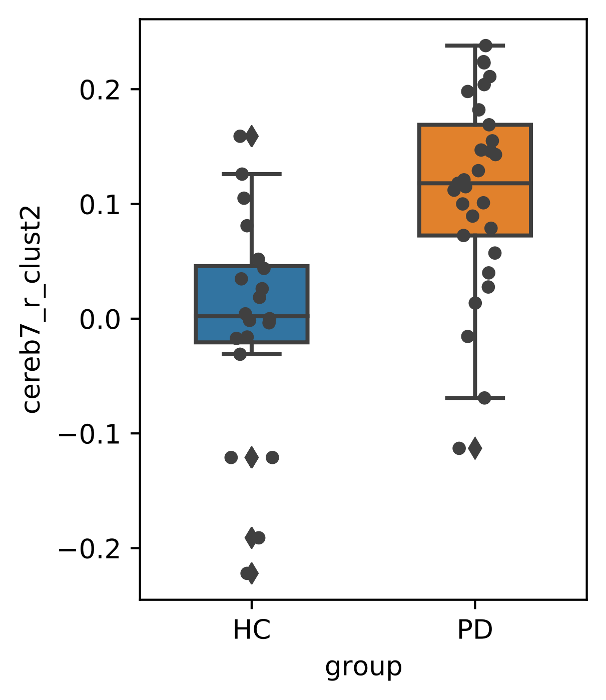

0.2
0.1
0.0
−0.1
−0.2
cereb7_r_clust2
HC
PD
group

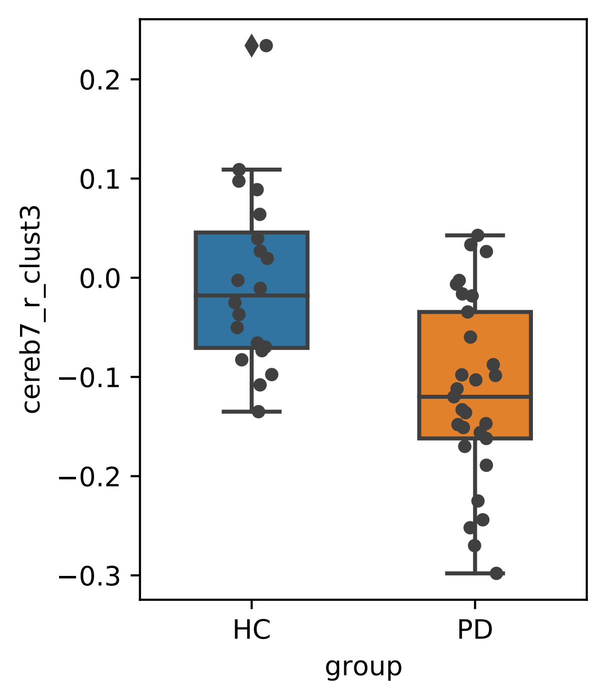

0.2
0.1
0.0
−0.1
−0.2
−0.3
cereb7_r_clust3
HC
PD
group

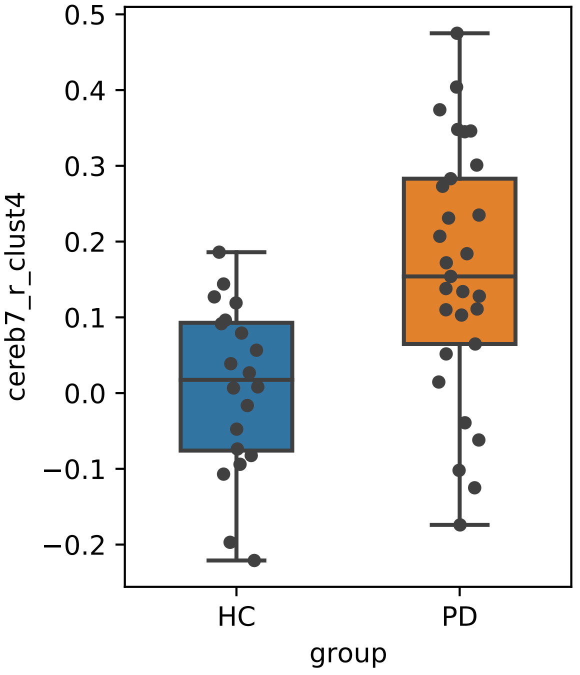
0.5
0.4
0.3
0.2
0.1
0.0
−0.1
−0.2
cereb7_r_clust4
HC
PD
group

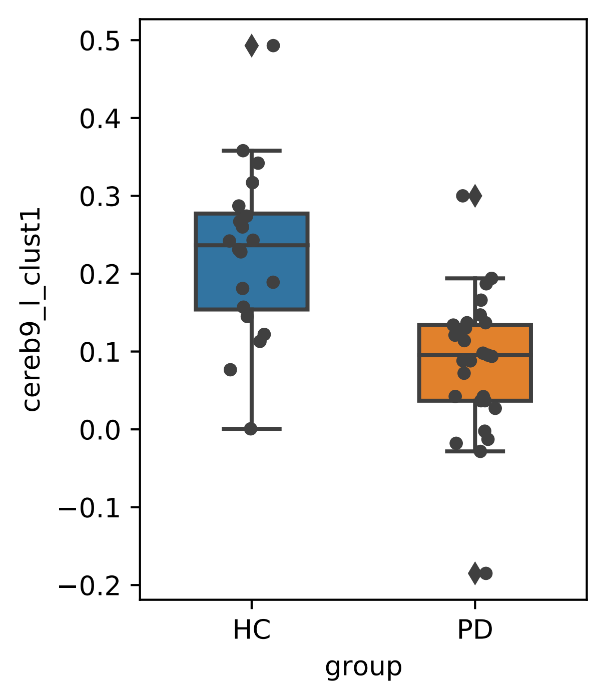
0.5
0.4
0.3
0.2
0.1
0.0
−0.1
−0.2
cereb9_l_clust1
HC
PD
group

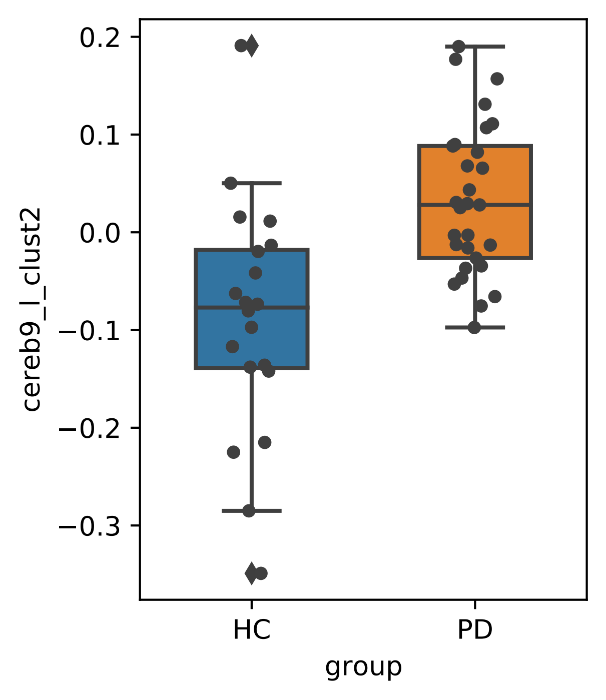

0.2
0.1
0.0
−0.1
−0.2
−0.3
cereb9_l_clust2
HC
PD
group

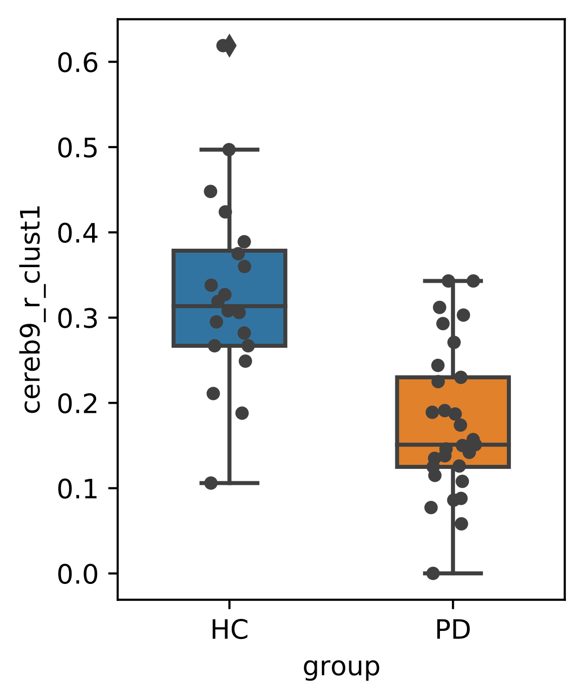

0.6
0.5
0.4
0.3
0.2
0.1
0.0
cereb9_r_clust1
HC
PD
group

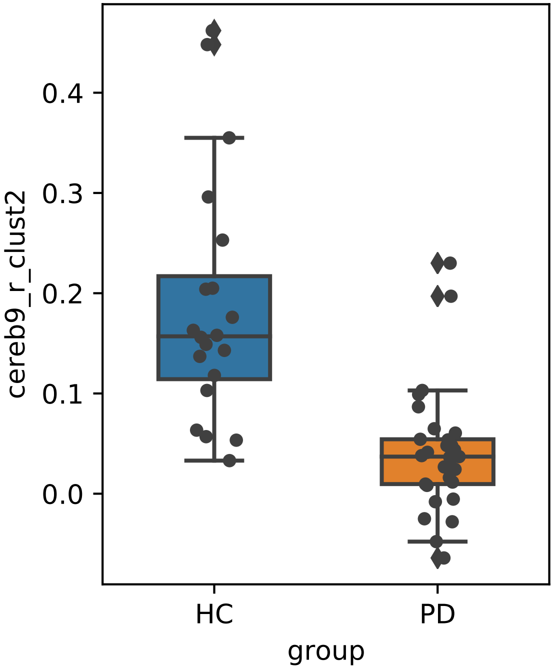

cereb9_r_clust2
0.4
0.3
0.2
0.1
0.0
HC
PD
group

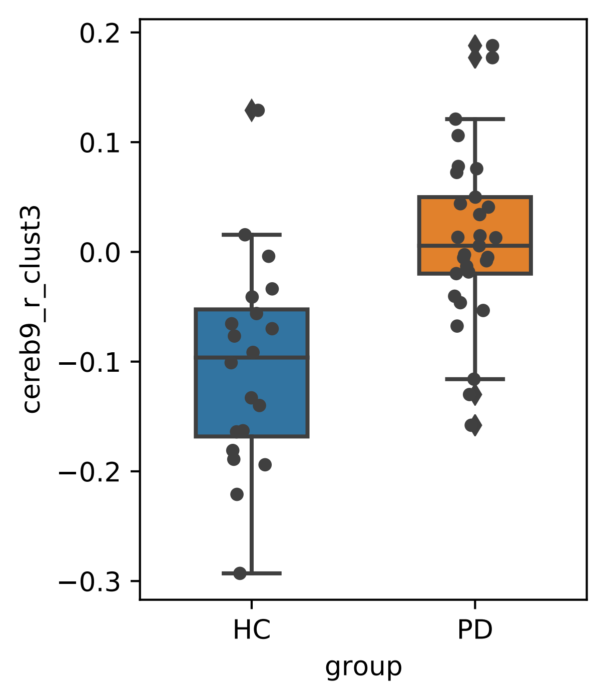

0.2
0.1
0.0
−0.1
−0.2
−0.3
cereb9_r_clust3
HC
PD
group

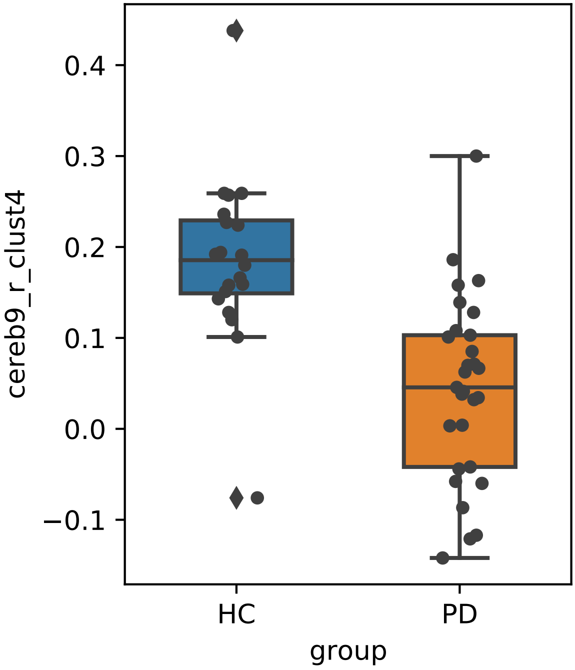

cereb9_r_clust4
0.4
0.3
0.2
0.1
0.0
−0.1
HC
PD
group

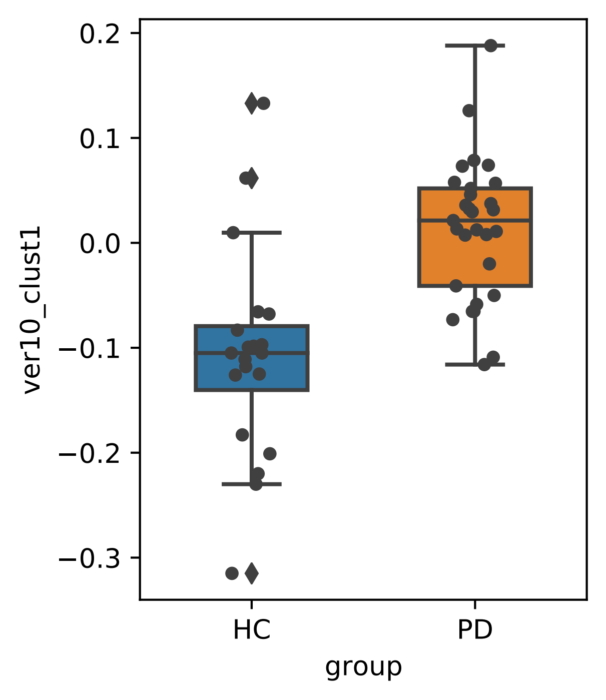
0.2
0.1
0.0
−0.1
−0.2
−0.3
ver10_clust1
HC
PD
group

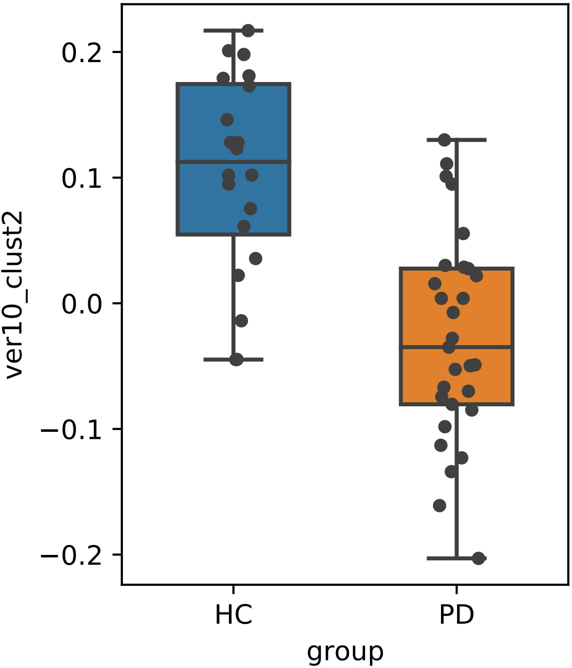

0.2
0.1
0.0
−0.1
−0.2
ver10_clust2
HC
PD
group

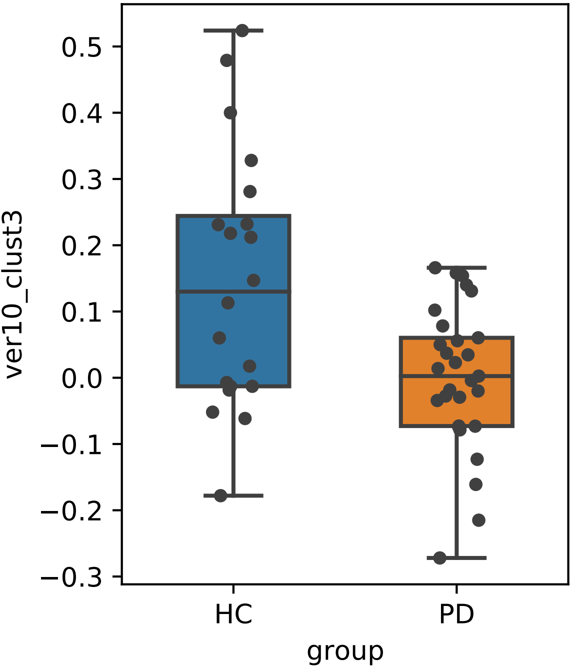

0.5
0.4
0.3
0.2
0.1
0.0
−0.1
−0.2
−0.3
ver10_clust3
HC
PD
group

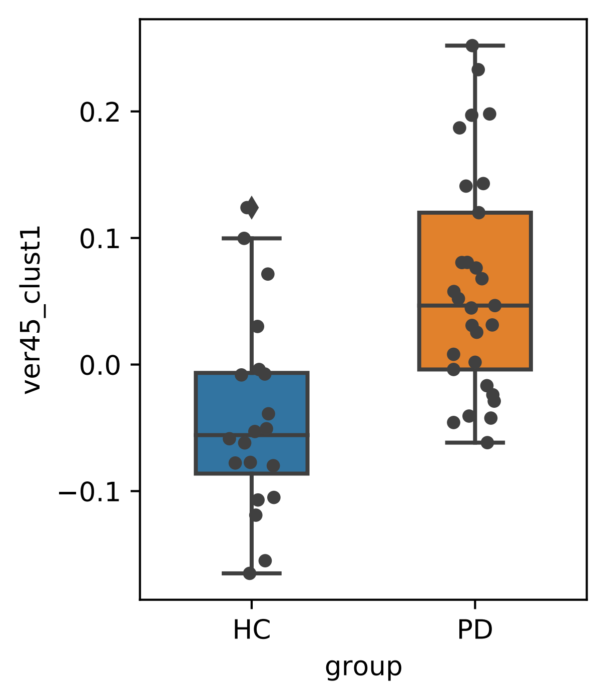

ver45_clust1
0.2
0.1
0.0
−0.1
HC
PD
group

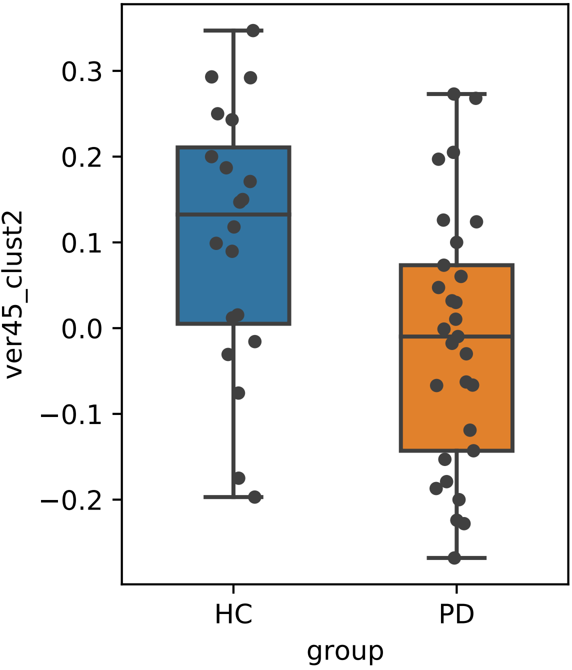

ver45_clust2
0.3
0.2
0.1
0.0
−0.1
−0.2
HC
PD
group

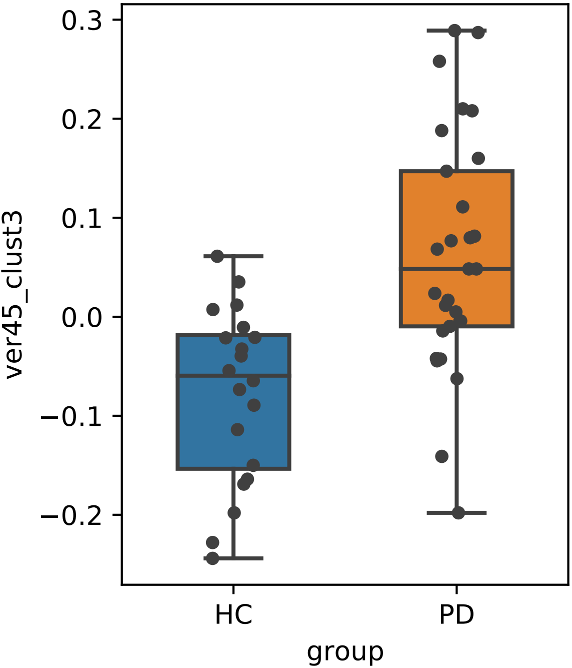

0.3
0.2
0.1
0.0
−0.1
−0.2
ver45_clust3
HC
PD
group

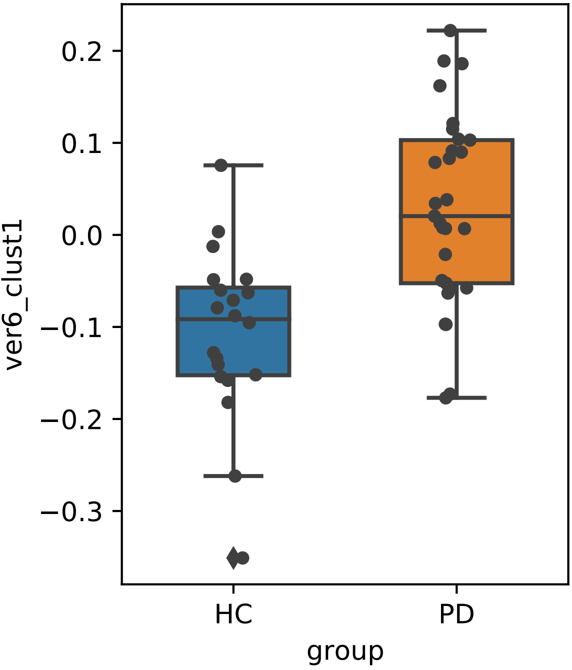
0.2
0.1
0.0
−0.1
−0.2
−0.3
ver6_clust1
HC
PD
group

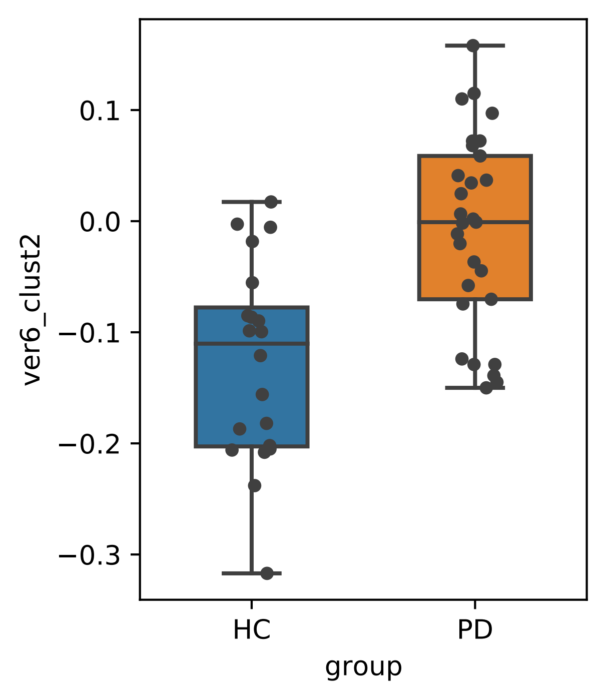

0.1
0.0
−0.1
−0.2
−0.3
ver6_clust2
HC
PD
group

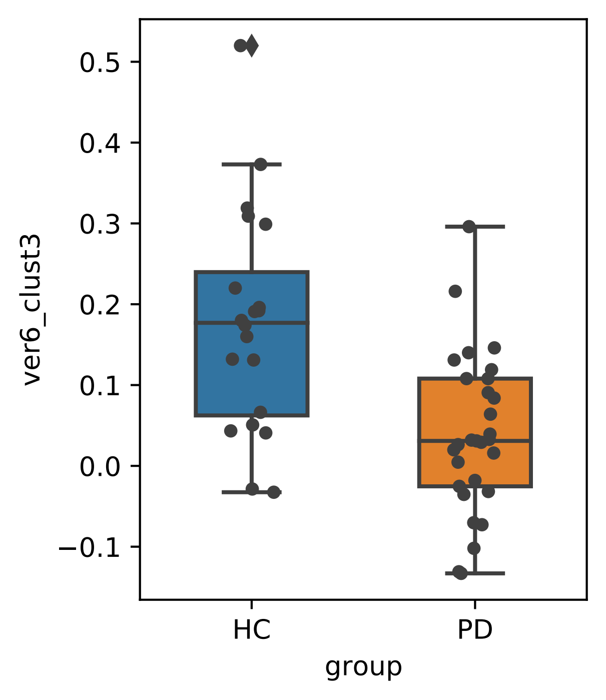
0.5
0.4
0.3
0.2
0.1
0.0
−0.1
ver6_clust3
HC
PD
group

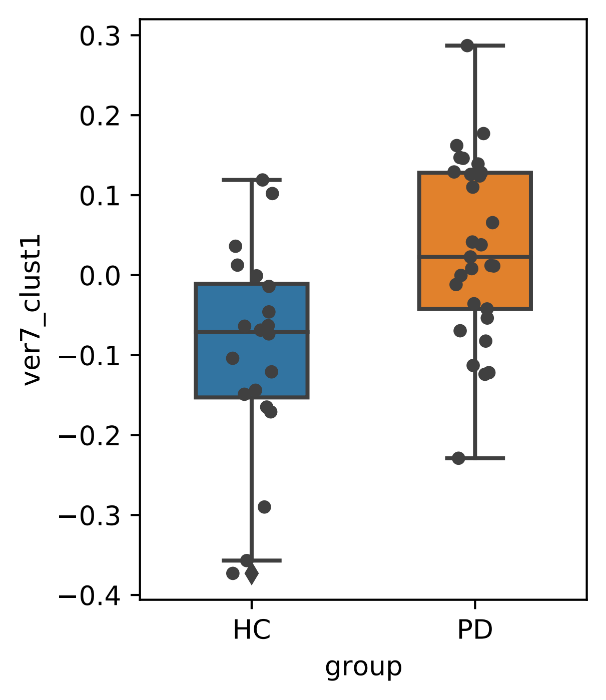

0.3
0.2
0.1
0.0
−0.1
−0.2
−0.3
−0.4
ver7_clust1
HC
PD
group

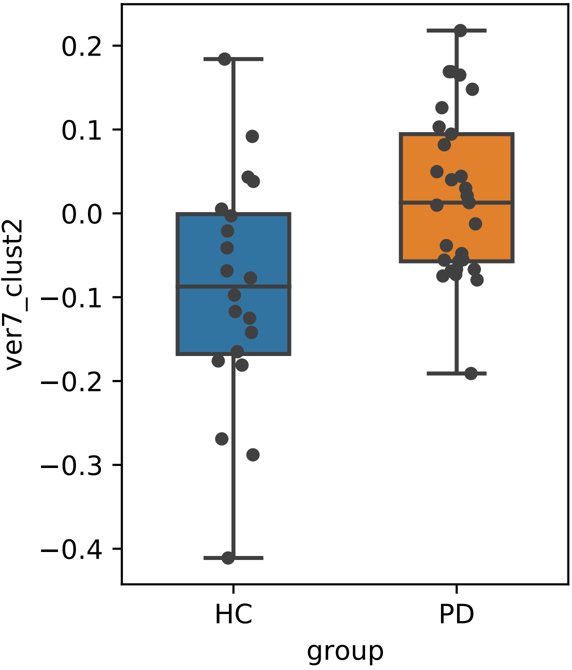

0.2
0.1
0.0
−0.1
−0.2
−0.3
−0.4
ver7_clust2
HC
PD
group

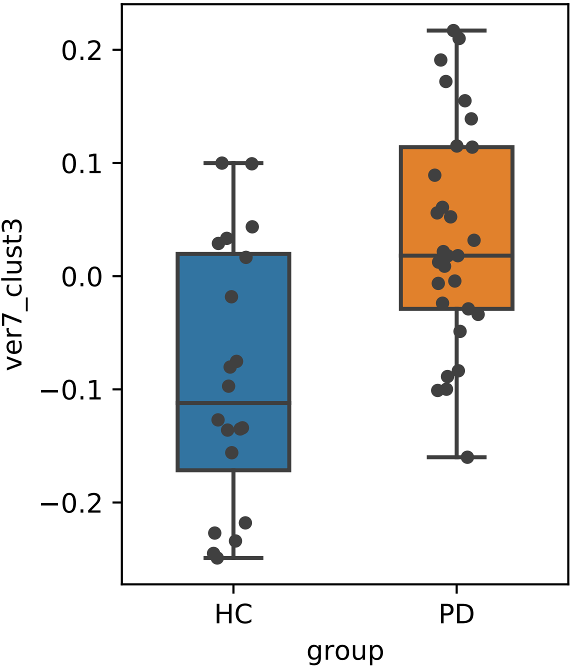

0.2
0.1
0.0
−0.1
−0.2
ver7_clust3
HC
PD
group

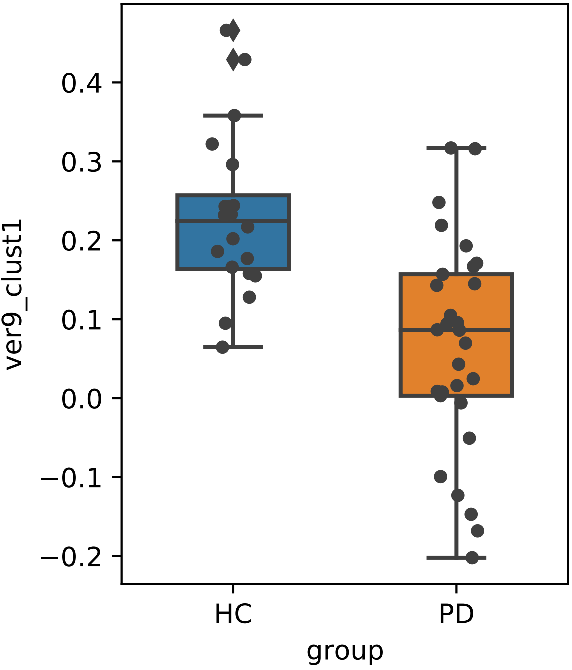

0.4
0.3
0.2
0.1
0.0
−0.1
−0.2
ver9_clust1
HC
PD
group

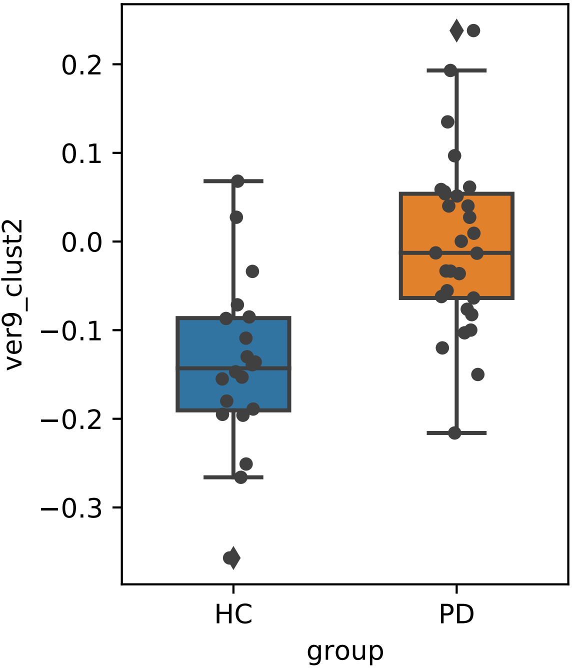
0.2
0.1
0.0
−0.1
−0.2
−0.3
ver9_clust2
HC
PD
group

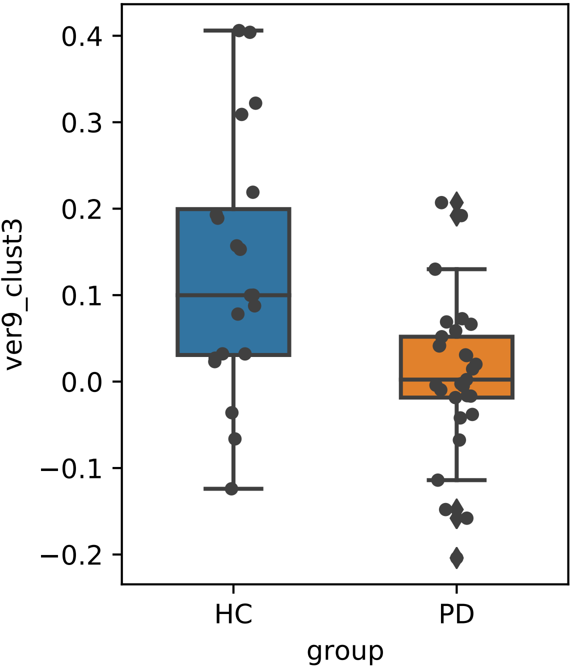
0.4
0.3
0.2
0.1
0.0
−0.1
−0.2
ver9_clust3
HC
PD
group
